# Stabilization of the Cart–Inverted-Pendulum System Using State-Feedback Pole-Independent MPC Controllers

**DOI:** 10.3390/s22010243

**Published:** 2021-12-29

**Authors:** Lotfi Messikh, El-Hadi Guechi, Sašo Blažič

**Affiliations:** 1 Laboratoire d’Automatique de Skikda (LAS), Département de Génie Électrique, Faculté de Technologie, Université 20 Août 1955, BP 26, Route El-Hadaeik, Skikda 21000, Algeria; l.messikh@univ-skikda.dz (L.M.); e.guechi@univ-skikda.dz (E.-H.G.); 2 Faculty of Electrical Engineering, University of Ljubljana, Tržaška 25, 1000 Ljubljana, Slovenia

**Keywords:** cart–inverted pendulum (CIP) system, explicit control scheme (ECS), cascade control scheme, model predictive control (MPC), coefficient diagram method (CDM), coincident pole placement method (CPP)

## Abstract

In this paper, a pole-independent, single-input, multi-output explicit linear MPC controller is proposed to stabilize the fourth-order cart–inverted-pendulum system around the desired equilibrium points. To circumvent an obvious stability problem, a generalized prediction model is proposed that yields an MPC controller with four tuning parameters. The first two parameters, namely the horizon time and the relative cart–pendulum weight factor, are automatically adjusted to ensure a priori prescribed system gain margin and fast pendulum response while the remaining two parameters, namely the pendulum and cart velocity weight factors, are maintained as free tuning parameters. The comparison of the proposed method with some optimal control methods in the absence of disturbance input shows an obvious advantage in the average peak efficiency in favor of the proposed SIMO MPC controller at the price of slightly reduced speed efficiency. Additionally, none of the compared controllers can achieve a system gain margin greater than 1.63, while the proposed one can go beyond that limit at the price of additional degradation in the speed efficiency.

## 1. Introduction

The cart–inverted pendulum (CIP) system that belongs to the class of fast single-input, multiple-output (SIMO), under-actuated systems and satisfies a set of complicated characteristics, such as fourth-order highly nonlinear dynamics, open-loop instability, state coupling, and non-minimum-phase (NMP) behavior, provides many challenging problems to standard and modern control techniques [1]. In the context of the CIP system stabilization, moving the cart from an initial position to a final destination while keeping the pendulum erected in the upright position has been extensively studied in the past, and many output-feedback and (static and dynamic) state-feedback control techniques have been developed to solve it. However, solving this task efficiently in the framework of linear static state-feedback control (SFC) to ensure prescribed system gain margin in addition to good time response behavior with pole-independent parameter tuning is a subject that still needs further investigation.

There are different types of control methods that have been applied to the inverted pendulum systems [1], including model predictive control (MPC) and non-MPC methods. With regard to the complicated characteristics of the inverted pendulum plants, the needed controlled system performance, and the limited control input effort resource, time-domain optimization techniques, such as the MPC [2,3,4,5,6], seem to be one of the most convenient ways to tackle the above control problem, especially when state and control input constraints are considered. The key feature of the MPC method is based on the following three successive steps [3]: (i) the explicit use of a model and system measurements to predict the future behavior of the controlled variables over a specified future time horizon, (ii) the calculation of a control sequence minimizing a cost function, and (iii) the application of the first control signal of the sequence for a given time before returning to step (i). MPC algorithms differ amongst themselves in the model used to represent the plant, the cost function to be minimized, the optimization method, and the adopted horizon time size and partition. Depending on the optimization problem underhand, they can lead to explicit or non-explicit control schemes. For fast, NMP, and under-actuated systems, such as the CIP system, the above design issues appear to be more challenging when dealing with the design of SIMO MPC controllers, especially if the stabilization requirements are to obtain (i) prescribed system gain margin, (ii) short CIP settling time with insignificant overshoot and undershoot, and (iii) reduced control effort. Examples of MPC and non-MPC methods are described in the next sections.

Based on the linearized CIP dynamics about the upright (unstable) equilibrium point and the linear MPC theory, many linear MPC control schemes have also been designed to solve the CIP stabilization problem. In [7,8], the concept of predictive pole placement was established, and the application of its intermittent linear quadratic formulation to an inverted pendulum was successfully realized in [9], showing good control performance. In [10], a mathematical model of the PS600 CIP system was derived and linearized. Then, a model predictive controller was designed on the basis of a linearized discrete model and a quadratic cost function. The controller was verified in both simulations and real-time experiments. In [11], a linear model predictive control with a quadratic cost function was designed and experimentally validated on a rotary inverted pendulum apparatus to study the effect of the input disturbance. In [12], a cascade MPC CIP stabilization controller was derived from the minimizing of two separate pendulum and cart-associated quadratic functions. The inner and outer controllers are tuned to obtain a double critically damped behavior for the inner and outer loops using a set of two adjusted parameters. Nonlinear MPC techniques have been also proposed to stabilize the CIP system [13,14,15,16]. Although these techniques have shown promising performance in tracking and stabilization problems, they are the most complicated control techniques in implementation due to the difficulties in obtaining an accurate nonlinear model, in adjusting the quadratic cost function weight factors, and in choosing or developing appropriate dedicated online optimization methods. The linear MPC technique shows fewer implementation difficulties, especially for explicit control schemes, in comparison to the nonlinear MPC at a price of reduced performance. Therefore, whenever linear MPC shows good performance for the considered problem, it usually is favored.

On the other hand, there are many proposed (MPC or non-MPC) linear explicit control schemes (ECS) to stabilize the CIP system, where the control input is evaluated directly in a single step and applied at the same time on the cart control input. These ECS include the coincident pole placement (CPP) [17], dominant pole placement (DPP) [18,19], two proportional-integral-derivatives (TPID) [20,21,22,23], and linear quadratic regulator (LQR) [22,23,24]. From the linear control theory point of view, the design task to satisfy some prescribed time (i.e., steady-state and transient) response performance may be regarded as a pole placement problem, especially when using CPP, DPP, and LQR methods. Once this problem is solved off-line by specifying the pole locations a priori with a pole-dependent method, as in the case of the CPP and DPP, or a posteriori with a pole-independent method, as in the LQR method, and the control parameter computation is also conducted off-line, the ECS-based control can be implemented easily in hardware and run in real-time. The design phase with controller tuning, which consists of determining the pole locations for the CPP and DPP and the weight matrices for the LQR, is the main challenge of such methods. It is usually performed by trial and error and depends on the designer’s experience [23]. In general, the use of such a tuning method to obtain some prescribed requirements not only takes much time but also does not guarantee that the best solution possible is found. To solve these difficulties, advanced numerical tuning algorithms, such as the genetic and particle swarm optimization algorithms have been proposed for automatic parameter tuning [24]. Notice that with a reduced set of parameters, the tuning difficulty becomes less problematic. Therefore, if a controller with few tuning parameters shows good performance for the considered problem, this can be seen as a huge practical advantage.

As can be seen, there are several interesting attempts to design linear static SFC for the CIP system stabilization in the form of MPC or non-MPC control schemes. MPC control schemes are more significant, because they can be considered optimal for a specified cost function. Notice that an ECS can be considered as an MPC method if there is a correspondence between the ECS gains and MPC parameters. The comparison, conducted in [25], between the MPC and LQR has shown that the MPC method is more suitable for the trajectory tracking task and smoothing in the control input, while the LQR is more convenient for fixed-value control and disturbance rejection, but it may generate adverse and rapid changes in the control signal. However, for both approaches, MPC and non-MPC, the presence of real NMP zeros in the cart part of the fourth-order linearized CIP transfer function limits the robustness performance and prevents the achievement of monotonic cart step responses. In order to obtain the best possible performance, the optimal choice of the SFC gains needs to be addressed. An important contribution in this line for pole-dependent non-MPC SFC controllers is attributed to the authors of [17], who proposed an analytical formula for optimal tuning of the SFC gains for the CIP system. In the derivation of the formula, the authors promote a priori a coincident-pole structure, which has a single tuning parameter (see Appendix C) for the closed-loop poles before maximizing the worst gain margin associated with the CIP output signals. In doing so, it is clear that the adopted configuration will impose an upper limit for the achievable system gain margin and prevent the controller from exploiting other possible pole configurations that may be more helpful in specifying a priori a prescribed gain margin and in reducing the impact of the closed-loop CIP zeros on its performance. In addition to the CIP stabilization problem, designing controllers that achieve non-overshooting/undershooting for all-pole systems (i.e., systems only having poles in their transfer functions) or minimum overshooting/undershooting for non-all-pole systems (i.e., systems having poles and zeroes in their transfer functions) have received considerable attention [26,27,28,29,30]. In [26], for example, the authors studied the overshoot of an all-pole fourth-order system with respect to the variation of pole locations, where the poles are parameterized with two damping ratios and two undamped natural frequencies. One of the main obtained results states that the system step input overshoot remains unchanged if the ratio of their two natural frequencies is kept constant. The other main result states that the overshoot of the considered system does not have monotonicity with respect to each damping ratio or their sum. The above statements no longer hold for our CIP system, which is a non-all-pole system. In this case, and due to the presence of a single real NMP zero, achieving the cart step response monotonicity with linear SFC is almost impossible [29,30], and it appears natural, as a possible objective, to turn toward the design of controllers that achieve as little undershoot/overshoot as possible while maintaining good robustness stability.

On the other hand, addressing the SFC gains tuning problem to achieve monotonic step responses with pole-independent tuning methods can be performed for all-pole systems using the well-known coefficient diagram method (CDM) [27,28,31]. In this method, controllers are designed via the assignment of the so-called characteristic ratios and generalized time constant (GTC), which may have a strong physical relationship with the damping (i.e., overshoot) and speed of response of the closed-loop system, respectively. With non-all-pole systems, the complexity of pole–zero interaction makes the standard CDM no longer valid. However, in the case of a non-all-pole system with one pair of jω-axis zeroes, the authors of [28] showed the possibility to obtain monotonic step response if the GTC is kept above a certain lower bound. For the CIP system, the cart system can be decomposed into the difference between a non-all-pole system with one pair of jω-axis zeroes and an all-pole system. Since it is impossible to ensure the cart monotonic step response, one can suggest as another possible objective to ensure the monotonicity of the non-all-pole system in the context of Manabe form, as is done in [28], without considering the impact of the remaining all-pole system (see Appendix A). In such a case, the robustness stability issue must be addressed, as well as the overshoot and undershoot response requirements (see Appendix A).

To the authors’ knowledge, the attempts to meet a prescribed system gain margin, short CIP settling time with insignificant overshoot and undershoot, and reduced control effort with pole-independent parameter tuning have not been considered before in the context of linear static SIMO MPC SFC control schemes. The proposed MPC controller is designed with a generalized prediction model to circumvent an obvious stability problem [32] and a quadratic cost function with four control parameters. The first two parameters, namely the horizon time and the relative cart–pendulum weight factor, are automatically adjusted to ensure a priori prescribed system gain margin and fast pendulum response, while the remaining two parameters, namely the pendulum and cart velocity weight factors, are maintained as free tuning parameters to tackle the damping and the reduced control effort problem. In contrast to the existing state-feedback methods to stabilize the CIP system, this work has the following main distinguishing features:

- From the theoretical point of view, a new robust pole-independent SIMO MPC controller with only two adjusted control parameters is proposed to solve the fourth-order CIP stabilization problem under full state availability, known CIP parameters, and a pendulum mass that is negligible in comparison to the cart mass. It is shown in this paper how to constraint the SFC controllers to ensure a priori a prescribed CIP system gain margin and how to constrain the MPC controller to have a fast pendulum response in comparison to the cart response, i.e., satisfying a two-time-scale structure in which the closed-loop pendulum subsystem responds faster than the closed-loop cart subsystem [33]. This contribution leads effectively to the reduction in the number of free tuned parameters from four to two. Another contribution, mainly inspired by the work of [17], is to derive optimal controllers that maximize the system gain margin for the standard CDM and cascade methods (see Appendix A, Appendix B and Appendix C). Finally, in order to obtain the optimal MPC weight factors and to allow performance comparison, the indices of speed and average peak efficiencies are introduced for characterizing the closed-loop CIP transient responses.
- From a practical point of view, for a prescribed system gain margin, the impact of the velocity cost function weight factors on the closed-loop system transient performance in the context of the proposed MPC method can be evaluated off-line, and the obtained trends can be easily clarified using two-dimensional graphical and contour plot representations. Some useful guidelines for rapid weighting factor adjustment are developed for the proposed method in the presence (or absence) of disturbance input. Additionally, standard and advanced numerical tuning algorithms can benefit from the obtained reduced two-dimensional space search to achieve global optimality for a given criterion. Such a situation helps in rapidly obtaining a solution to the parameter tuning problem and also helps in checking easily its optimality.

The paper is organized as follows. Section 2 defines the CIP models and states the problem under consideration. Section 3 deals with the SIMO MPC controller design. Section 4 provides simulations results, and finally, Section 5 concludes this paper.

## 2. CIP Models and Problem Statement

### 2.1. Nonlinear Inverted Pendulum Dynamics

**Figure 1:**
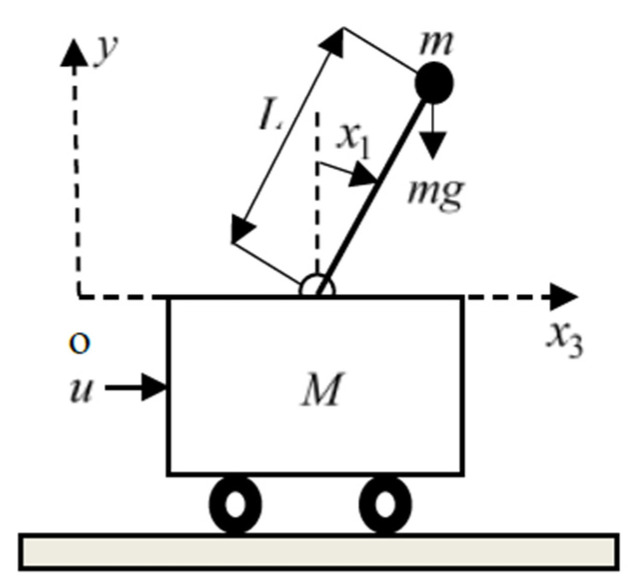
The inverted pendulum system.

The CIP system consists of a cart and a rigid rod pendulum with a pivot mounted on the top of the cart, as shown in Figure 1. Under the action of the horizontal force that is regarded as the control input u(t), the cart moves left or right on a one-dimensional bounded track, whereas the pendulum swings in the vertical plane determined by the track. It is assumed that no friction exists in the system between the cart and the track or between the cart and the pendulum. The cart is characterized by a mass M, and the pendulum is characterized by a length L of the mass-less rod and a point mass m located at the free end of the pendulum rod.

To describe the dynamics, a state vector $x={\left(x_{1},x_{2},x_{3},x_{4}\right)}^{T}$ is used, where the state signals $x_{1}$, $x_{2}$, $x_{3}$, and $x_{4}$ are the pendulum angle (measured clockwise w.r.t the upward vertical), the angular velocity of the pendulum, the cart position, and the cart velocity, respectively. Using Newton’s laws, it is possible to derive and describe the dynamics of the CIP system explicitly by the following fourth-order SIMO under-actuated and highly nonlinear dynamical system (see Appendix D):

(1) $$\begin{array}{l} {\dot{x}}_{1}=x_{2}\\ {\dot{x}}_{2}=-\frac{uL^{-1}\cos x_{1}-\left(M+m\right)gL^{-1}\sin x_{1}+mx_{2}^{2}\sin x_{1}\cos x_{1}}{M+m-m\cos^{2}x_{1}}\\ {\dot{x}}_{3}=x_{4}\\ {\dot{x}}_{4}=+\frac{u-mg\sin x_{1}\cos x_{1}+mLx_{2}^{2}\sin x_{1}}{M+m-m\cos^{2}x_{1}} \end{array}.$$

Notice that, in the above model, the pendulum and cart accelerations are driven with opposite direction input control forces over the upper half-plane of the pendulum angle. This makes our stabilization task more challenging.

### 2.2. Generalized Linear Prediction Model

Under the three assumptions taken from [12], the assumption of the light mass at the end of the rod, and the control input domination assumption

(2) $$\sin\;x_{1}\approx x_{1};\;\;\;\;\;\;\cos\;x_{1}\approx 1;\;\;\;\;\;\;gL^{-1}>>x_{2}^{2};\;\;\;\;\;\;M>>m;\;\;\;\;\;\;mg\left|x_{1}\right|<<\left|u\right|,$$

the nonlinear dynamical model (1) can be approximated by the following linear model:

(3) $$\left[\begin{array}{c} {\dot{x}}_{1}\\ {\dot{x}}_{2}\\ {\dot{x}}_{3}\\ {\dot{x}}_{4} \end{array}\right]=\left[\begin{array}{cccc} 0 & 1 & 0 & 0\\ a_{1} & 0 & 0 & 0\\ 0 & 0 & 0 & 1\\ 0 & 0 & 0 & 0 \end{array}\right]\left[\begin{array}{c} x_{1}\\ x_{2}\\ x_{3}\\ x_{4} \end{array}\right]+\left[\begin{array}{c} 0\\ -b_{1}\\ 0\\ +b_{2} \end{array}\right]u,$$

with

(4) $$\begin{array}{l} a_{1}=+gL^{-1};\;\;\;\;\;\;\;\;\;\;\;b_{1}=\frac{1}{ML};\;\;\;\;\;\;\;\;\;b_{2}=\frac{1}{M}\;\;\\ gb_{1}=a_{1}b_{2} \end{array}.$$

Notice that the parameters $a_{1}$, $b_{1}$, and $b_{2}$ are positive. It will be shown later in the paper that all the assumptions given in (2) are indeed satisfied. From (3), one can deduce the following unstable transfer functions:

(5) $$\begin{array}{l} H_{1}\left(s\right)=\frac{X_{1}\left(s\right)}{U\left(s\right)}=\frac{-b_{1}}{s^{2}-a_{1}}\\ H_{3}\left(s\right)=\frac{X_{3}\left(s\right)}{U\left(s\right)}=\frac{+b_{2}}{s^{2}} \end{array}.$$

Let us now define the following generalized linear CIP model with k∈{−1,+1}, which can be seen as a generalization or modification of model (3):

(6) $$\left[\begin{array}{c} {\dot{x}}_{1}\\ {\dot{x}}_{2}\\ {\dot{y}}_{3}\\ {\dot{y}}_{4} \end{array}\right]=\left[\begin{array}{cccc} 0 & 1 & 0 & 0\\ a_{1} & 0 & 0 & 0\\ 0 & 0 & 0 & 1\\ 0 & 0 & 0 & 0 \end{array}\right]\left[\begin{array}{c} x_{1}\\ x_{2}\\ y_{3}\\ y_{4} \end{array}\right]+\left[\begin{array}{c} 0\\ -b_{1}\\ 0\\ +kb_{2} \end{array}\right]u,$$

where the pendulum subsystem model remains unchanged when compared to the original model (3) while the cart subsystem model is substituted by a fictitious one. This last subsystem, with the new states $y_{3}$ and $y_{4}$, is driven at each instant t with the control input ku. Using the above-adopted model (6), and assuming $y_{3}=x_{3}$ and $y_{4}=x_{4}$ at the instant t, we define the following pendulum and cart generalized prediction models:

(7) $$\begin{array}{l} {\hat{x}}_{1}\;\left(t+h\right)=A_{1}x_{1}\left(t\right)+B_{1}x_{2}\left(t\right)\;-E_{1}u\\ {\hat{x}}_{2}\left(t+h\right)=A_{2}x_{1}\left(t\right)+B_{2}x_{2}\left(t\right)\;-E_{2}u\\ {\hat{y}}_{3}\;\left(t+h\right)=x_{3}\left(t\right)\;+h\;x_{4}\left(t\right)+kE_{3}u\\ {\hat{y}}_{4}\left(t+h\right)=x_{4}\left(t\right)+kE_{4}u \end{array},$$

where h is a positive constant horizon time. If u(t)=u is constant on the interval [t,t+h], then by solving (6) and using the last equality of (4), we obtain (7) with the following parameters:

(8) $$\begin{array}{l} A_{1}=\cosh\left(a_{1}^{1/2}h\right)\;\;\;\;\;\;\\ A_{2}=a_{1}^{1/2}\sinh\left(a_{1}^{1/2}h\right)\\ B_{1}=a_{1}^{-1/2}\sinh\left(a_{1}^{1/2}h\right)\\ B_{2}=\cosh\left(a_{1}^{1/2}h\right)\\ E_{1}=2a_{1}^{-1}b_{1}\sinh^{2}\left(a_{1}^{1/2}h/2\right)\\ E_{2}=a_{1}^{-1/2}b_{1}\sinh\left(a_{1}^{1/2}h\right)\\ E_{3}=0.5b_{2}\;h^{2}\\ E_{4}=b_{2}\;h \end{array}.$$

Notice that the parameters $A_{1}$, $A_{2}$, $B_{1}$, $B_{2}$, $E_{1}$, $E_{2}$, $E_{3}$, and $E_{4}$ are positive.

### 2.3. Problem Statement

For the CIP system stabilization, we are interested in using the following static state-feedback control law (SFC):

(9) $$\begin{array}{ll} u\left(t\right) & =+Nx\left(t\right)-N_{3}x_{3d}\\ & =+N_{1}x_{1}\left(t\right)+N_{2}x_{2}\left(t\right)+N_{3}\left[x_{3}\left(t\right)-x_{3d}\right]+N_{4}x_{4}\left(t\right) \end{array},$$

where $N=\left(N_{1},N_{2},N_{3},N_{4}\right)$ is the control gain vector to be determined, and $x_{3d}$ is the constant cart reference position. The main result of this paper is how to simplify the control tuning based on the good robustness properties of the control system and the optimized two-time controller structure. The controllability of the linear system (3) ensures the existence of a set of SFC having the form (9) that can achieve the stabilization of the CIP system in the vicinity of the unstable equilibrium point. Combining (5) and the Laplace transform of (9) yields:

(10) $$\begin{array}{l} F_{1}\left(s\right)=\frac{X_{1}\left(s\right)}{X_{3d}\left(s\right)}=\frac{+b_{1}N_{3}s^{2}}{P\left(s\right)}\\ F_{3}\left(s\right)=\frac{X_{3}\left(s\right)}{X_{3d}\left(s\right)}=\frac{-b_{2}N_{3}\left(s^{2}-a_{1}\right)}{P\left(s\right)}\\ F_{u}\left(s\right)=\frac{U\left(s\right)}{X_{3d}\left(s\right)}=\frac{-N_{3}s^{2}\left(s^{2}-a_{1}\right)}{P\left(s\right)}\\ P\left(s\right)=s^{4}+\left(b_{1}N_{2}-b_{2}N_{4}\right)\;s^{3}+\left(b_{1}N_{1}-b_{2}N_{3}-a_{1}\right)\;s^{2}+a_{1}b_{2}N_{4}\;s+a_{1}b_{2}N_{3} \end{array}.$$

**Figure 2:**
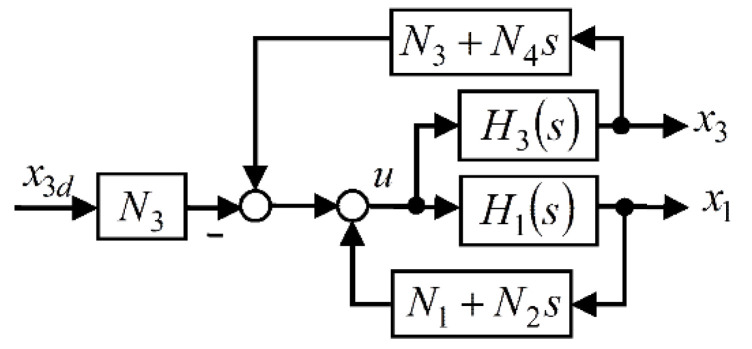
State-feedback linearized CIP control system.

The obtained linearized closed-loop CIP system is of the fourth order and has the basic configuration shown in Figure 2. Obviously, each controller belonging to the class (9) can be interpreted as the combination of two PD controllers (one for the pendulum subsystem and the other for the cart subsystem).

**Remark 1.**  *The fourth-order transfer functions (10) must at least be stable. The Routh necessary and sufficient stability conditions for such a system are given in (11). From these conditions and the positivity of the parameters (4), it follows that the controller gains*$\left(N_{1},N_{2},N_{3},N_{4}\right)$*must be positive*.

(11) $$\begin{array}{l} b_{1}N_{2}-b_{2}N_{4}>0\\ b_{1}N_{1}-b_{2}N_{3}-a_{1}>0\\ N_{4}>0\\ N_{3}>0\\ b_{1}N_{2}-b_{2}N_{4}>\frac{N_{4}}{N_{2}}\frac{g}{\frac{N_{1}}{N_{2}}-\frac{N_{3}}{N_{4}}-\frac{a_{1}}{b_{1}N_{2}}} \end{array}.$$

**Remark 2.**  *The cart transfer function *$F_{3}\left(s\right)$*has two real opposite zeroes*, $z_{1,2}=\pm a_{1}^{1/2}$. *The single real NMP zero*, $z_{2}=+a_{1}^{1/2}$, *provokes the appearance of an undesirable initial undershoot in the cart step response. The amplitude of this undershoot grows to infinity if the settling time is reduced to 0* [30]. *This behavior therefore places a limitation on the cart speed of response. Overshoot in the above response is another undesirable effect that may be reduced with the undershoot phenomenon if an appropriate selection of SFC controller gains is conducted*.

**Remark 3.**  *From (10), it can be seen that the pendulum and cart transfer functions*$F_{1}\left(s\right)$*and*$F_{3}\left(s\right)$*have fixed zeroes and adjustable poles. Thus, whatever the method used to determine the SFC gains, it is always considered as a pole placement method. In the sequel, we shall refer to the SFC control method (9) as pole-dependent and pole-independent if its gain tuning method specifies a priori and a posteriori the closed-loop pole locations of (10), respectively*.

**Figure 3:**
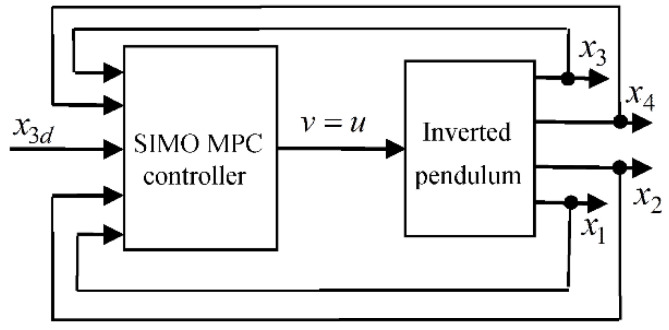
The proposed CIP SIMO MPC control system.

In this paper, we investigate a subclass of SFC controllers (9), where the designed controller carries all the key features of SIMO MPC controllers and can be implemented in an ECS fashion. As it is depicted in Figure 3, the considered controller receives as its inputs the cart reference position, $x_{3d}$, and the state vector, $x=\left(x_{1},x_{2},x_{3},x_{4}\right)$, and produces as its single output the control signal, v, which is fed directly to the control input u of the CIP system. In our investigation, we need to consider the following assumption.

Assumption 1:

- the state vector $x=\left(x_{1},x_{2},x_{3},x_{4}\right)$ is measurable;
- the parameters $\left(M,m,L,x_{3d}\right)$ are known constants; and
- the set of hypotheses in Equation (2) is satisfied.

Under Assumption 1, the CIP stabilization problem may be formulated in two steps as follows. In the first step, restrict the class of SFC controllers so as to satisfy the following property:

- Property 1: The SFC controller (9) ensures robust stability with a prescribed closed-loop gain margin, $GM_{\min}$.

In the second step, and under the above gain margin constraint and the generalized prediction model (7) and (8), design a pole-independent SIMO MPC controller such that the following properties are satisfied:

- Property 2: The closed-loop CIP system satisfies a two-time-scale structure in which the closed-loop pendulum subsystem responds faster than the closed-loop cart subsystem [33].
- Property 3: In the absence of disturbance input, the controller ensures low cart settling time T>0 without excessive peaking (undershoot and overshoot) phenomenon in the CIP input and output responses.

## 3. SIMO MPC Controller Design

To design robust SIMO MPC controllers, we first developed Equation (23), associated with the SFC gain margin property, using the stability conditions (11) and the prescribed gain margin, $GM_{\min}$. Then, Property 1 holds when the above-mentioned equation is satisfied. Next, based on the generalized prediction model (7–8) and the quadratic cost function (24) that is characterized by a set of MPC parameters $\varphi =\left(h,\rho_{1},r,\rho_{2}\right)$, i.e., the horizon time and the positive weight factors to be defined later, we establish the explicit relationship (26) between φ and N. Finally, we propose a pole-independent tuning method that relies primarily on choosing the parameter set φ of the cost function as a starting point of the optimal control. This method leads to determining the pole locations a posteriori, i.e., at its final stage. Notice that the controller design developed hereafter uses the linearized CIP model (3) in the neighborhood of the equilibrium point, where it is assumed to be justified at least from the approximation point of view. The validity of the adopted design methods is demonstrated from the control point of view in Section 4, where the nonlinear model (1) is used instead of its linearized version.

### 3.1. Closed-Loop SGM Constraint

Let us consider the following change of variables:

(12) $$\begin{array}{l} N_{1}=\alpha_{12}N_{2}\\ N_{3}=\alpha_{34}N_{4}\\ \alpha =\alpha_{12}-\alpha_{34} \end{array},$$

where $\alpha_{12}>0$ and $\alpha_{34}>0$. Introducing (12) in the last condition of (11) gives:

(13) $$b_{1}N_{2}>\left[1+\frac{b_{1}}{b_{2}}\frac{g}{b_{1}N_{2}\alpha -a_{1}}\right]b_{2}N_{4}.$$

This means that there exists a critical gain $N_{4}$ that depends on the fixed value of $N_{2}$. The critical positive gain $b_{2}N_{4i}>0$ at which the system becomes marginally stable is:

(14) $$b_{2}N_{4i}=\frac{b_{1}N_{2}}{1+\frac{b_{1}}{b_{2}}\frac{g}{b_{1}N_{2}\alpha -a_{1}}}>b_{2}N_{4}.$$

From (14), we define the cart-loop gain margin as follows:

(15) $$GM_{C}=\frac{b_{2}N_{4i}}{b_{2}N_{4}}=\frac{b_{1}N_{2}}{b_{2}N_{4}}\frac{1}{1+\frac{a_{1}}{b_{1}N_{2}\alpha -a_{1}}}.$$

On the other hand, the critical positive gains $b_{1}N_{2i}\alpha >0$ and $y_{i}=b_{1}N_{2i}\alpha -a_{1}$ at which the system becomes marginally stable is deduced from (13) as the positive solutions of the following system of equations:

(16) $$\begin{array}{l} y_{i}^{2}+\left[a_{1}-b_{2}N_{4}\alpha \right]y_{i}-gb_{1}N_{4}\alpha =0\\ y_{i}=b_{1}N_{2i}\alpha -a_{1} \end{array},$$

with

(17) $$\begin{array}{l} y_{i}=\frac{1}{2}\left[b_{2}N_{4}\alpha -a_{1}\right]+\frac{1}{2}\sqrt{{\left[b_{2}N_{4}\alpha -a_{1}\right]}^{2}+4gb_{1}N_{4}\alpha }>0\\ b_{1}N_{2i}\alpha =y_{i}+a_{1} \end{array}.$$

Using the last equality $gb_{1}=a_{1}b_{2}$ of (4), (17) reduces to:

(18) $$\begin{array}{l} y_{i}=b_{2}N_{4}\alpha >0\\ b_{1}N_{2i}\alpha =b_{2}N_{4}\alpha +a_{1} \end{array}.$$

From (18), we define the pendulum-loop gain margin as follows:

(19) $$GM_{P}=\frac{b_{1}N_{2}\alpha }{b_{1}N_{2i}\alpha }=\frac{b_{1}N_{2}}{b_{2}N_{4}}\frac{1}{1+\frac{a_{1}}{b_{2}N_{4}\alpha }}.$$

Taking into account the worst case, the system gain margin index can be defined as follows [17]:

(20) $$\begin{array}{l} GM=\min\left(GM_{P},GM_{C}\right)\\ GM=\left\{ \begin{array}{l} GM_{C}\;\mathrm{if}\;\;\left(b_{1}N_{2}-b_{2}N_{4}\right)\alpha <a_{1}\\ GM_{P}\;\mathrm{if}\;\left(b_{1}N_{2}-b_{2}N_{4}\right)\alpha \geq a_{1} \end{array}\right. \end{array}.$$

Since the gain margins, $GM_{C}$ and $GM_{P}$, must be greater than 1, from (16) and (19), we deduce, respectively:

(21) $$\begin{array}{l} \left(b_{1}N_{2}-b_{2}N_{4}\right)\alpha =a_{1}+\left(GM_{C}-1\right)\alpha b_{2}N_{4}\geq a_{1}\\ \left(b_{1}N_{2}-b_{2}N_{4}\right)\alpha =GM_{P}a_{1}+\left(GM_{P}-1\right)\alpha b_{2}N_{4}\geq a_{1} \end{array}.$$

Regarding (21), the system gain margin (20) reduces to:

(22) $$GM=GM_{P}=\frac{b_{1}N_{2}}{b_{2}N_{4}}\frac{1}{1+\frac{a_{1}}{b_{2}N_{4}\alpha }}.$$

The following remark states some of the obvious properties of the closed-loop system gain margin.

**Remark 4.**  *Consider the fourth-order closed-loop system in (10) with the change of variables (12) and the system gain margin (22). Then, the following properties hold: (i) with fixed parameters*α and $b_{1}N_{2}$, GM *decreases with the increase in* $b_{2}N_{4}$; *(ii) with fixed parameters* α *and* $b_{2}N_{4}$, GM *increases with the increase in* $b_{1}N_{2}$; *and (iii) with fixed parameters* $b_{1}N_{2}$ *and* $b_{2}N_{4}$, GM *remains unchanged for any value* γ *if* $N_{1}$ *and* $N_{3}$ *are increased by the amounts* $N_{2}\gamma$ *and* $N_{4}\gamma$, *respectively*.

**Remark 5.**  *Given the value of*$GM_{\min}$*and using Equation (22), Property 1 can be satisfied under the following constraint*:

(23) $$\left(b_{1}N_{2}-GM_{\min}b_{2}N_{4}\right)\alpha =GM_{\min}a_{1},$$

*where*α*is defined in (12).*

### 3.2. Deriving the SIMO MPC Control Law

To derive the SIMO MPC control law, we first adopt the generalized linear prediction model (7) and (8) as a prediction model for the CIP system. Then, we consider the following quadratic cost function:

(24) $$\begin{array}{l} J_{MPC}\left(t,h\right)=\frac{1}{2}e_{1}^{2}\left(t+h\right)+\frac{1}{2}\rho_{1}e_{2}^{2}\left(t+h\right)+\frac{1}{2}re_{3}^{2}\left(t+h\right)+\frac{1}{2}r\rho_{2}e_{4}^{2}\left(t+h\right)\\ e_{1}\left(t+h\right)=0-{\hat{x}}_{1}\left(t+h\right)\\ e_{2}\left(t+h\right)=0-{\hat{x}}_{2}\left(t+h\right)\\ e_{3}\left(t+h\right)=x_{3d}-{\hat{y}}_{3}\left(t+h\right)\\ e_{4}\left(t+h\right)={\dot{x}}_{3d}-{\hat{y}}_{4}\left(t+h\right)=0-{\hat{y}}_{4}\left(t+h\right) \end{array},$$

where $\varphi =\left(h,\rho_{1},r,\rho_{2}\right)\in {ℜ}^{+4}$ is the set of positive MPC parameters, $e_{1}\left(t+h\right)$ and $e_{2}\left(t+h\right)$ are the predicted pendulum angle and velocity errors, while $e_{3}\left(t+h\right)$ and $e_{4}\left(t+h\right)$ are the predicted cart position and velocity errors. Now, given the cart destination $x_{3d}$ and the prediction models (7), we obtain the optimal input $v_{0}$, which minimizes the value of the quadratic cost function (24). Recall that the references associated with $x_{1}$, $x_{2}$, and $x_{4}$ are assumed to be zero. Substituting (7) into (24) and setting the gradient of $J_{MPC}\left(t,h\right)$ with respect to v to zero yields:

(25) $$v_{0}\left(t\right)\;\;\;\;\;=+N_{1}x_{1}\left(t\right)+N_{2}x_{2}\left(t\right)+N_{3}\left[x_{3}\left(t\right)-x_{3d}\right]+N_{4}x_{4}\left(t\right),$$

with the SIMO MPC controller gains given below:

(26) $$\begin{array}{l} N_{1}=D^{-1}A_{1}E_{1}+D^{-1}\rho_{1}A_{2}E_{2}\\ N_{2}=D^{-1}B_{1}E_{1}+D^{-1}\rho_{1}B_{2}E_{2}\\ N_{3}=-D^{-1}r\times kE_{3}\\ N_{4}=-D^{-1}r\times k\left(hE_{3}+\rho_{2}E_{4}\right)\\ D=E_{1}^{2}+\rho_{1}E_{2}^{2}+r\times k^{2}\left(E_{3}^{2}+\rho_{2}E_{4}^{2}\right) \end{array}.$$

Notice that the positivity of $\left(\rho_{1},r,r\rho_{2}\right)$ together with the positivity of (8) imply the positivity of $N_{1}$ and $N_{2}$ in addition to the fact that the gains $N_{3}$ and $N_{4}$ have an undefined sign that follows the one associated with the value of k.

**Remark 6.**  *Since the signs of*$N_{3}$*and*$N_{4}$*are identical to the sign of*k, *the designed SIMO MPC controller that is defined by (23) and (24) does not ensure the stability condition (11) when using the trivial prediction model, i.e., the generalized prediction model (10) with*k=+1. *To circumvent such a drawback, we put*k=−1*in the above model. This modification is equivalent to the substitution of the original cart subsystem model by another cart subsystem model that is driven at each instant and from the same state point with an opposite control input sign. The modification of the cost function while keeping the trivial prediction model is another option to solve the encountered stability problem. However, this option is more complicated and is not considered in this paper.*

### 3.3. Time-Scale Structure Constraint

**Figure 4:**
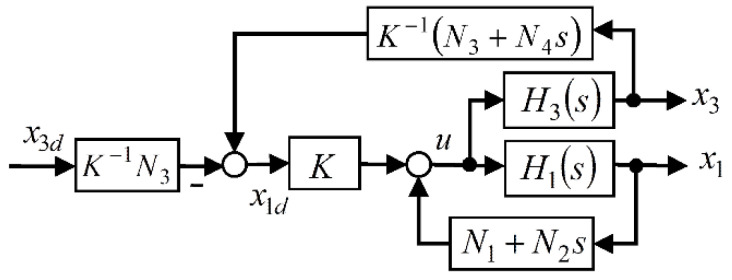
Equivalent state-feedback linearized CIP control system.

Here, we are interested in designing a SIMO MPC controller that ensures Property 2, i.e., a two-time-scale structure, without using a priori targeted closed-loop pole locations. To this end, the CIP control system of Figure 2 is transformed to the configuration of Figure 4, where a virtual reference $x_{1d}$ and a new gain K are introduced for the closed-loop pendulum subsystem. The pendulum transfer function to the considered reference and the value of K that leads to a unity static gain are given by:

(27) $$\begin{array}{l} F_{1F}\left(s\right)=\frac{X_{1}\left(s\right)}{X_{1d}\left(s\right)}=\frac{-Kb_{1}}{s^{2}+b_{1}N_{2}s+b_{1}N_{1}-a_{1}}\\ K=-N_{1}+a_{1}b_{1}^{-1}=-N_{1}+Mg \end{array}.$$

The GTC associated with (10) and (27) are given, respectively, by:

(28) $$\begin{array}{l} \tau_{C}=\frac{a_{1}b_{2}N_{4}}{a_{1}b_{2}N_{3}}=\frac{N_{4}}{N_{3}}=\frac{1}{\alpha_{34}}\\ \tau_{P}=\frac{b_{1}N_{2}}{b_{1}N_{1}-a_{1}}=\frac{1}{\alpha_{12}-MgN_{2}^{-1}}>\tau_{PL}=\frac{1}{\alpha_{12}} \end{array},$$

where $\tau_{PL}$ is a lower bound for the pendulum GTC.

For the sake of developing a general tuning method that is valid for a large class of CIP systems, let us define the following normalized parameters:

(29) $$\begin{array}{l} h_{0}=a_{1}^{1/2}h\;\;\;\;\;\;\;\;\;\;\;\;\;\;\;\rho_{10}=a_{1}\rho_{1}\;\;\;\;\;\;\;\;\;\;\;\;\;\;\;\rho_{20}=a_{1}\rho_{2}\\ r_{0}=a_{1}^{-2}g^{2}r\;\;\;\;\;\;\;\;\;\;\;\;r_{12}=a_{1}^{-1/2}\alpha_{12}\;\;\;\;\;\;\;\;\;r_{34}=a_{1}^{-1/2}\alpha_{34}\\ \varphi_{0}=\left(h_{0},\rho_{10},r_{0},\rho_{20}\right) \end{array},$$

where $\varphi_{0}$ is the set of normalized positive MPC cost function parameters. Substituting (29) into (28) and (26), we obtain:

(30) $$\begin{array}{l} r_{12}=\frac{N_{10}}{N_{20}}=a_{1}^{-1/2}\tau_{PL}^{-1}\\ r_{34}=\frac{N_{30}}{N_{40}}=a_{1}^{-1/2}\tau_{C}^{-1} \end{array},$$

(31) $$\begin{array}{l} N_{1}=a_{1}b_{1}^{-1}N_{10}D_{0}^{-1}\\ N_{2}=a_{1}^{1/2}b_{1}^{-1}N_{20}D_{0}^{-1}\\ N_{3}=a_{1}b_{2}^{-1}N_{30}D_{0}^{-1}\\ N_{4}=a_{1}^{1/2}b_{2}^{-1}N_{40}D_{0}^{-1} \end{array},$$

with

(32) $$\begin{array}{ll} N_{10} & =2\sinh^{2}\left(h_{0}/2\right)\cosh\left(h_{0}\right)+\rho_{10}\sinh^{2}\left(h_{0}\right)\\ & =4\sinh^{4}\left(h_{0}/2\right)+2\sinh^{2}\left(h_{0}/2\right)+\rho_{10}\sinh^{2}\left(h_{0}\right)\\ N_{20} & =2\sinh\left(h_{0}\right)\sinh^{2}\left(h_{0}/2\right)+\rho_{10}\cosh\left(h_{0}\right)\sinh\left(h_{0}\right)\\ N_{30} & =0.5r_{0}h_{0}^{2}\\ N_{40} & =r_{0}\left(0.5h_{0}^{2}+\rho_{20}\right)\;h_{0}\\ D_{0} & =4\sinh^{4}\left(h_{0}/2\right)+\rho_{10}\sinh^{2}\left(h_{0}\right)+r_{0}\left(0.25h_{0}^{2}+\rho_{20}\right)\;h_{0}^{2} \end{array}.$$

Now, combining (30) and (32) yields:

(33) $$r_{12}=a_{1}^{-1/2}\tau_{PL}^{-1}=\frac{4\sinh^{4}\left(h_{0}/2\right)+2\sinh^{2}\left(h_{0}/2\right)+\rho_{10}\sinh^{2}\left(h_{0}\right)}{2\sinh\left(h_{0}\right)\sinh^{2}\left(h_{0}/2\right)+\rho_{10}\cosh\left(h_{0}\right)\sinh\left(h_{0}\right)}.$$

In order to speed the pendulum response, we plan to maximize (33) by a proper choice of the horizon time. More precisely, for a given positive value $\rho_{10}$, setting the gradient of $r_{12}$ with respect to $h_{0}$ to zero yields:

(34) $$h_{0}=\ln\left[\frac{1+\rho_{10}+\rho_{10}^{2}+\sqrt{\rho_{10}\left(1+2\rho_{10}\right)\left(2+\rho_{10}\right)}}{1-\rho_{10}^{2}}\right]\;\;.$$

**Figure 5:**
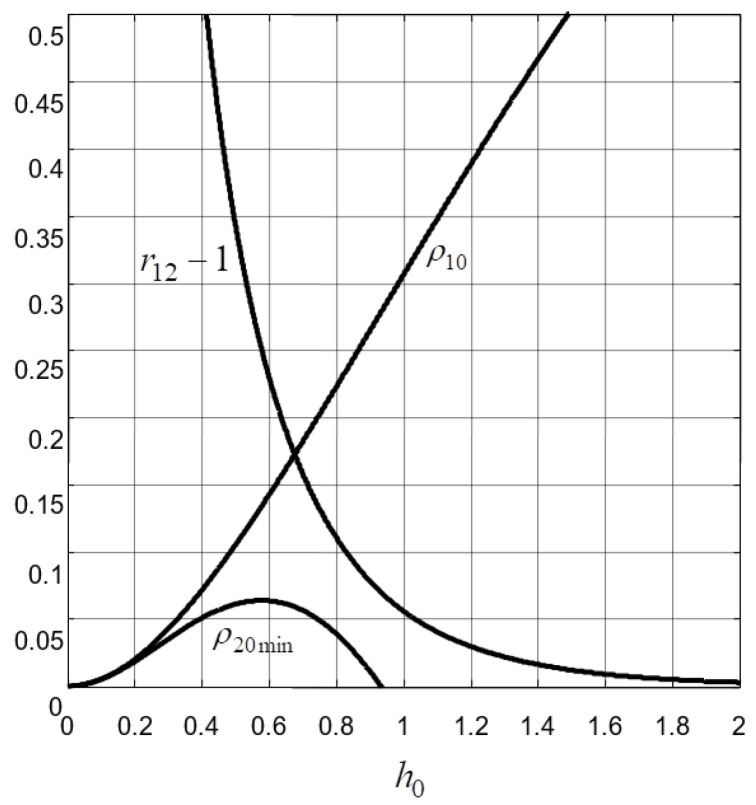
Evolution of ($r_{12}$, $\rho_{10}$, $\rho_{20\min}$) versus $h_{0}$.

From (34) and Figure 5, it is clear that the parameter $\rho_{10}$ increases with the increase in $h_{0}$ and satisfies $0<\rho_{10}<1$. Concerning the amount $r_{12}$, it is obvious that this parameter decreases toward 1 with the increase in $h_{0}$.

Now, introducing the expressions of $\alpha_{12}$ and $\alpha_{34}$ from (29) together with the expressions of $N_{2}$ and $N_{4}$ from (31) in (23) gives

(35) $$\left(N_{20}-GM_{\min}N_{40}\right)\left(r_{12}-r_{34}\right)=GM_{\min}D_{0}.$$

Substituting the expressions of $N_{40}$ and $D_{0}$ from (32) in (35) and solving for $r_{0}$ gives:

(36) $$r_{0}=\frac{GM_{\min}^{-1}N_{20}\left(r_{12}-r_{34}\right)-4\sinh^{4}\left(h_{0}/2\right)-\rho_{10}\sinh^{2}\left(h_{0}\right)}{\left(r_{12}-r_{34}\right){\left(0.5h_{0}^{2}+\rho_{20}\right)}^{}\;h_{0}+\left(0.25h_{0}^{2}+\rho_{20}\right){\;}^{}h_{0}^{2}}.$$

Since $h_{0}$ and $\rho_{10}$ are linked by the relationship (34) and the fact that $r_{0}$ and $\left(h_{0},\rho_{10},\rho_{20}\right)$ are linked by the relationship (36), we only need to specify two parameters among the elements of the set $\varphi_{0}$ to achieve the controller design. In our case, we shall fine-tune the weight factor vector $\rho_{0}=\left(\rho_{10},\rho_{20}\right)$ inside an appropriate domain. We have already shown that $0<\rho_{10}<1$, and the interval associated with $\rho_{20}$ remains to be determined. To this end, it is reasonable to satisfy Property 2 by assuming that the GTC of the closed-loop cart subsystem is lower than the GTC of the pendulum subsystem. According to (30) and (32) and the fact that $r_{0}>0$, this leads us to impose the constraint:

(37) $$0<r_{34}=\frac{0.5h_{0}}{0.5h_{0}^{2}+\rho_{20}\;}<r_{12},$$

from which we obtain:

(38) $$\begin{array}{l} \rho_{20\min}<\rho_{20}<\infty \\ \rho_{20\min}=\frac{1}{2}\left(r_{12}^{-1}-h_{0}\right)h_{0} \end{array}.$$

A lower bound $\rho_{20\min}$, which is shown in Figure 5 as a function of $h_{0}$, is then imposed to $\rho_{20}$ to ensure Property 2.

Now, let us define a set of five indices to evaluate the CIP transient response performance: $P_{x1}\left(\rho_{0}\right)$, the maximum absolute value of the pendulum angle response; $V_{x3}\left(\rho_{0}\right)$, the cart response overshoot; $D_{x3}\left(\rho_{0}\right)$, the cart response undershoot; $P_{u}\left(\rho_{0}\right)$, the maximum absolute value of the control input signal; and $t_{cs}\left(\rho_{0}\right)$, the cart settling time at 5%. To derive general comments on the behavior of the CIP system, it is useful to introduce the change variable $s=a_{1}^{1/2}z$ and the gains (31) in (10) to obtain the following transfer functions:

(39) $$\begin{array}{l} F_{1}\left(z\right)=L^{-1}\frac{N_{30}D_{0}^{-1}z^{2}}{P\left(z\right)}\\ F_{3}\left(z\right)=\frac{-N_{30}D_{0}^{-1}\left(z^{2}-1\right)}{P\left(z\right)}\\ F_{u}(s)=-MgL^{-1}\frac{N_{30}D_{0}^{-1}z^{2}\left(z^{2}-1\right)}{P\left(z\right)}=\\ P\left(z\right)=z^{4}+\left(N_{20}D_{0}^{-1}-N_{40}D_{0}^{-1}\right)\;z^{3}+\left(N_{10}D_{0}^{-1}-N_{30}D_{0}^{-1}-1\right)\;z^{2}+N_{40}D_{0}^{-1}\;z+N_{30}D_{0}^{-1} \end{array}.$$

With the above processing, all the considered CIP transient response characteristics remain unchanged, except the one associated with the modified (normalized) cart settling time $t_{csn}$, which is now linked to the original cart settling time $t_{cs}$ by the relationship:

(40) $$t_{csn}=a_{1}^{1/2}t_{cs}.$$

Regarding the transfer functions (39), it appears naturally useful to normalize the indices associated with the pendulum angle response and control input signal as follows:

(41) $$\begin{array}{l} P_{x1n}=LP_{x1}\\ P_{un}=P_{u}/\left(MgL^{-1}\right) \end{array}.$$

Now, we are ready to summarize in Algorithm 1 how to design the proposed SIMO controller and how to obtain the closed-loop linearized CIP performance when the system gain margin $GM_{\min}$ and the weight vector $\rho_{0}=\left(\rho_{10},\rho_{20}\right)$ are chosen.


**Algorithm 1: Controller design and closed-loop linearized CIP system evaluation.**

Set the worst system gain margin, *GM*_min_, and choose the weight vector $\rho_{0}=\left(\rho_{10},\rho_{20}\right)$;Evaluate $\left(h_{0},r_{12},r_{34},r_{0},\rho_{20\min}\right)$ using (34), (33), (37), (36), and (38), respectively;If *r*_0_ < 0 or *ρ*_20_ ≤ *ρ*_20 min_, go to step 7;Evaluate (*N*_10_, *N*_20_, *N*_30_, *N*_40_, *D*_0_) using (32);Evaluate the step responses of (39) with (*L*,*Mg*) = (1,1);Evaluate the indices ($P_{x1n},V_{x3n},D_{x3n},P_{un},t_{csn}$) from the obtained step responses;End.


### 3.4. Parameter Tuning

In this section, parameter tuning for the weight vector $\rho_{0}$ is performed to ensure the validity of Property 3. To this end, the peaking and speed constraints need to be considered simultaneously. To solve this problem, a time-domain optimization technique that involves tackling two issues, namely the choice of the performance criterion and the mean to optimize it, is proposed hereafter. For the first issue, we have found it useful, regarding the desirable Property 3, to define two proposed sound and scaled-free indices. These indices are the speed efficiency $S_{E}\left(\rho_{0}\right)$ and the average peak efficiency $S_{E}\left(\rho_{0}\right)$ and are defined for a given standard (reference) state-feedback method (SSF) and our control method as follows:

(42) $$\begin{array}{l} S_{E}\left(\rho_{0}\right)=100\frac{P_{4,SSF}}{P_{4}\left(\rho_{0}\right)+P_{4,SSF}}\\ G_{E}\left(\rho_{0}\right)=\frac{100}{3}\sum_{i=1}^{3}\frac{P_{i,SSF}}{P_{i}\left(\rho_{0}\right)+P_{i,SSF}} \end{array},$$

where $P_{1}\left(\rho_{0}\right)=P_{x1}\left(\rho_{0}\right)$, $P_{2}\left(\rho_{0}\right)=P_{x3}\left(\rho_{0}\right)$, $P_{3}\left(\rho_{0}\right)=P_{u}\left(\rho_{0}\right)$, and $P_{4}\left(\rho_{0}\right)=t_{cs}\left(\rho_{0}\right)$. The index $P_{i,SSF}$ has the same interpretation as the index $P_{i}\left(\rho_{0}\right)$, with the proposed control method replaced by the SSF one. Obviously, an efficiency index greater than 50% indicates that the proposed control method outperforms the SSF method; otherwise, degradation in the performance of our method in comparison to the SSF one is noted. Then, taking into account the worst efficiency case, we may formulate the setting problem as a maximin optimization model as follows:

(43) $$\begin{array}{l} \left(\rho_{10,g},\rho_{20,g}\right)=\underset{\left(\rho_{10},\rho_{20}\right)}{\arg}\underset{\begin{array}{l} 0\leq \rho_{10}\leq \rho_{10,\max}\\ 0\leq \rho_{20}\leq \rho_{20,\max} \end{array}}{\max}\;J_{1}\left(\rho_{0}\right)\\ J_{1}\left(\rho_{0}\right)=\;\min\;\left(S_{E}\left(\rho_{0}\right),\lambda^{-1}G_{E}\left(\rho_{0}\right)\right) \end{array},$$

where λ=0 if speed efficiency is the only concern, and λ=1 if peaking and speed efficiency are both considered in the optimization. It should be noted that (43) is a continuous nonlinear optimization model with a highly nonlinear (possibly discontinuous) objective function $J_{1}\left(\rho_{0}\right)$, and it is difficult to know a priori whether such a function is unimodal or multimodal before starting the optimization. To avoid erroneous solutions, problem (43) has to be solved to global optimality in the considered parameter space domain. Then, to solve the second issue, we opt for global optimization using an exhaustive search over the domain that is defined by $0\leq \rho_{10}\leq \rho_{10,\max}$ and $0\leq \rho_{20}\leq \rho_{20,\max}$. This is interesting, since there are only two tuning parameters.

## 4. Numerical Simulations

Numerical simulations are conducted in several separate sections to show the potential of the proposed pole-independent SIMO MPC controller (SIM) and its advantage in comparison to the pole-independent standard CDM controller (CDM), the pole-dependent CASC MPC controller (CAS), and the coincident pole controller (CPP). The definitions of the CDM, CAS, and CPP controllers are given in Appendix A, Appendix B and Appendix C, respectively. These controllers are chosen for their efficiencies and for the reduced number of adjusted parameters, which leads to yield, without significant difficulty, a guarantee of the overall best performance for each tuned controller. Based on the linearized CIP system, we begin the numerical simulation in Section 4.1 with the establishment of some guidelines that help in choosing the SIM weight factors for a prescribed system gain margin in the absence of disturbance input. These guidelines are quite general, since they can be applied to any linear CIP model of the form (3), as long as Assumption 1 stays satisfied. Additionally, they are very useful since they inform us how to tune the SIM weight factors to get some desirable transient closed-loop linearized CIP system performance. Next, to make a fair comparison, we tune, for a given physical CIP system, the best possible CDM, CAS, and CPP controller so as to obtain a maximum system gain margin for each tuned controller. For the SIM controller, the system gain margin is set a priori as the best one so far obtained by the above controllers, and the SIM weight factors are tuned according to (43). In Section 4.3 and Section 4.4, we compare the obtained controllers on the nonlinear CIP system in the absence and presence of disturbance input.

### 4.1. Guidelines for Weighting Factor Adjustment

**Figure 6:**
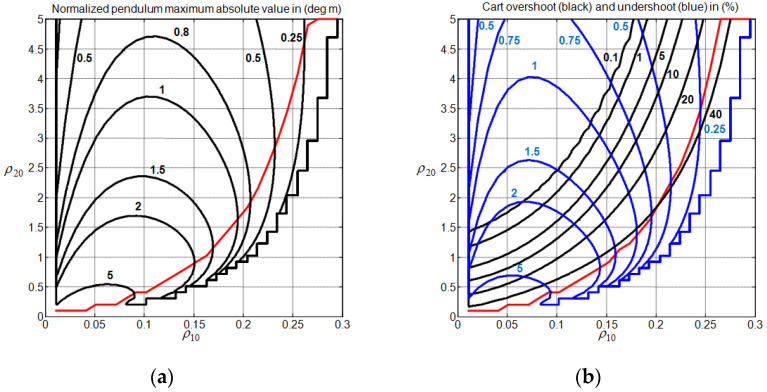
CIP control performance $GM_{\min}=1.63$. (**a**) Normalized pendulum maximum absolute value index; (**b**) cart overshoot and undershoot indices.

**Figure 7:**
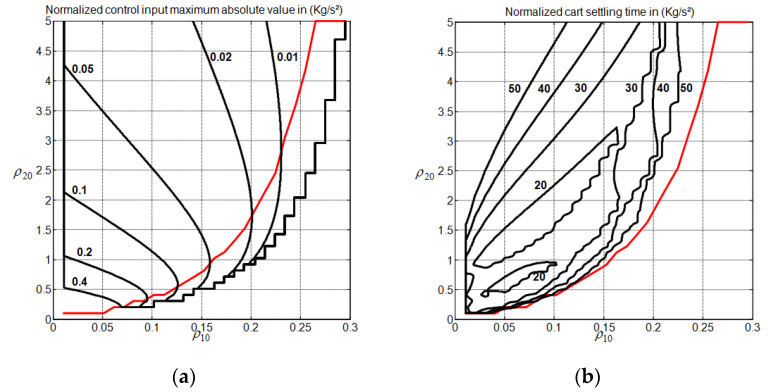
CIP control performance for $GM_{\min}=1.63$. (**a**) Normalized control input maximum absolute value index; (**b**) normalized cart settling time index.

To obtain some insights into how to choose the weight vector $\rho_{0}=\left(\rho_{10},\rho_{20}\right)$ for different system gain margin values in the absence of disturbance input, we conducted 50,000 simulations with Algorithm 1 (obtained by using a coarse grid discretization with 50 points for $\rho_{10}$ in the interval $0<\rho_{10}<0.5$, 50 points for $\rho_{20}$ in the interval $0<\rho_{20}<5$, and 20 points for $GM_{\min}$ in the interval $1<GM_{\min}<3$). Figure 6 and Figure 7 show the obtained contour plots for the considered transient response characteristics for $GM_{\min}=1.63$. To generate the contours, we constructed a grid interpolant using the Matlab function “griddedInterpolant” with the “pchip” option and interpolate the considered index with $10^{-3}$ spacing. To avoid dummy solutions, only the contours that have more than $10^{3}$ points were retained.

From Figure 6 and Figure 7, one can observe that the choice of the weight factors cannot be performed independently to address the peaking phenomenon and the cart speed of response issues simultaneously. Additionally, a strong correlation between the contours of $P_{x1n}$ and $D_{x3}$ is noticed. For the peaking phenomenon, it is clearly seen that the increase in $\rho_{20}$ while maintaining $\rho_{10}$ constant reduces without ambiguity the cart overshoot $V_{x3}\left(\rho_{0}\right)$. Ensuring a non-overshooting behavior for the cart subsystem thus appears possible with a high enough value of $\rho_{20}$. For a constant $\rho_{10}$, the peaking phenomenon indices, namely $P_{x1n}$, $D_{x3}$, and $P_{un}$, are only reduced when $\rho_{0}$ is located above their associated red line frontiers, as shown in Figure 6 and Figure 7. The evaluation of these frontiers is performed without considering the interpolation processing. Ensuring a reduced undershooting behavior for the cart subsystem appears to be possible with the increase in $\rho_{20}$. In addition to that, one can also clearly observe that the increase in $\rho_{10}$ while maintaining $\rho_{20}$ constant contribute, without ambiguity, to reduce the control input effort $P_{un}$ and to increase the cart overshoot $V_{x3}\left(\rho_{0}\right)$, as long as the value of $\rho_{10}$ still far enough from the frontier that it defines the validity of the MPC controller (see step 3 of Algorithm 1). For a constant $\rho_{20}$, the increase in $\rho_{10}$ may lead to an increase in $P_{x1n}$ and $D_{x3}$. Concerning the cart speed of response, there are two separate regions, i.e., the upper region UR and the lower region LR, where the settling time can be reduced below the value 20. These regions are located above the red line, where the increase in $\rho_{20}$ while maintaining $\rho_{10}$ constant reduces the peaking phenomenon. Table 1 shows the associated region frontier locations and transient performance intervals. Obviously, choosing $\rho_{0}$ in the UR appears to be more recommended than choosing it in the LR.

When the cart speed is the only concern, i.e., when using the proposed tuning method (43) with λ=0, there is no need to select an SSF method, and the best-achieved cart settling time, $t_{csn,b}$, and its associated weight factor, $\rho_{10,b}$, are defined as follows:

(44) $$\begin{array}{l} t_{csn,b}=\underset{\begin{array}{l} 0\leq \rho_{10}\leq 0.5\\ \;\;0\leq \rho_{20}\leq 5 \end{array}}{\min}t_{csn}\left(\rho_{10},\rho_{20},GM_{\min}\right)\\ \rho_{10,b}=\underset{\rho_{10}}{\arg}\underset{\begin{array}{l} 0\leq \rho_{10}\leq 0.5\\ \;0\leq \rho_{20}\leq 5 \end{array}}{\min}t_{csn}\left(\rho_{10},\rho_{20},GM_{\min}\right) \end{array}.$$

**Figure 8:**
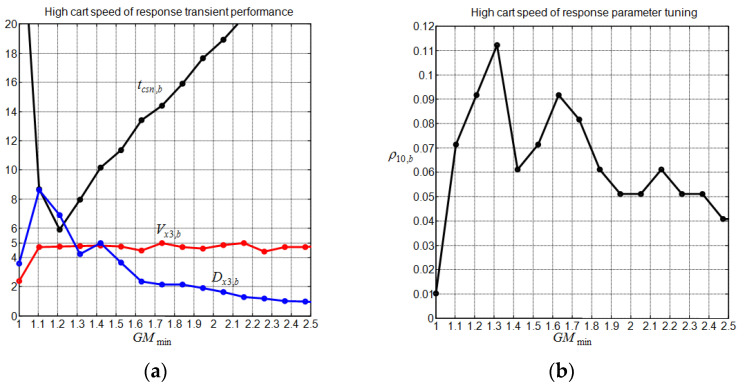
Impact of the gain margin on the cart transient of response. (**a**) Optimal cart control subsystem performance versus gain margin; (**b**) weight factor versus gain margin setting for high cart speed of response.

Figure 8 shows the above indices together with the cart overshoot and undershoot as a function of the prescribed system gain margin. It is clearly seen that the best-achieved cart settling time is obtained with $GM_{\min}\approx 1.2$; additionally, the increase in $GM_{\min}$ above this value leads to an increase in the optimal cart settling time and a reduction in the cart undershoot. The cart overshoot remains between 4 and 5%, while the optimal weight factor $\rho_{10,b}$ remains, in all studied cases, under 0.12. As we shall see in the next section, the considered controllers for the comparison task do not exceed a system gain margin of 1.63, while our SIM controller can go beyond this limit, as is indicated in Figure 8.

### 4.2. Disturbance-Free Parameter Tuning

**Figure 9:**
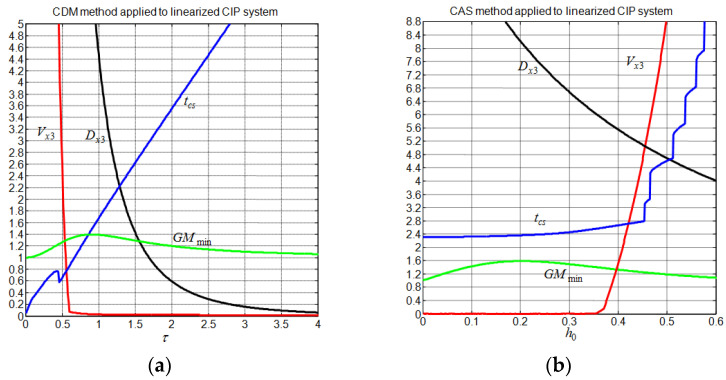
Evolution of the system gain margin and the cart response performance. (**a**) CDM method; (**b**) CAS method.

**Figure 10:**
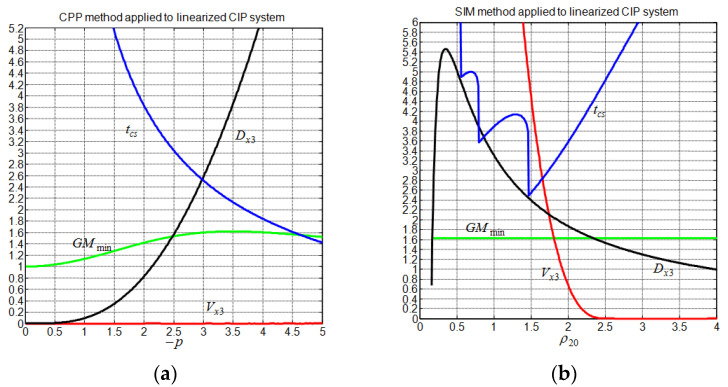
Evolution of the system gain margin and the cart response performance. (**a**) CPP method; (**b**) SIM method.

Now, let us consider the nonlinear CIP system (1) with a set of physical parameters M=2.4 kg, m=0.23 kg, L=0.36 m, $g=9.81\;{m/s}^{2}$, and a cart track length limited between ±0.5m [23]. Since the behavior of the linearized CIP system is considered quite similar to the behavior of the nonlinear CIP system in the vicinity of the equilibrium point, the tuning of the controllers is thus based only on the linearized system. Figure 9 and Figure 10 show the evolution of the system gain margin together with the associated linearized cart transient performance for the considered controllers. In contrast to non-undershooting cart response behavior, ensuring a non-overshooting cart response behavior is a feasible objective for all controllers. Reducing the cart response undershoot can be undertaken at the price of an increase in the cart settling time and/or deterioration in the system gain margin performance. Regarding the system gain margin trend, it is seen that the system gain margin is limited by about 1.4 for the CDM method and by about 1.6 for the CAS and CPP methods. For these methods, dependencies are typically observed between the system gain margin and the tuning parameters, which render them less flexible. Now, following [17], we retain the best tuning parameter for the CDM, CAS, and CPP as those that maximize the system gain margin. The gains of these controllers and the resulting linear CIP system transient performance are summarized in Table 2 and Table 3, respectively. The transient performances are obtained for zero initial conditions and a cart step $x_{3d}=0.1$.

**Figure 11:**
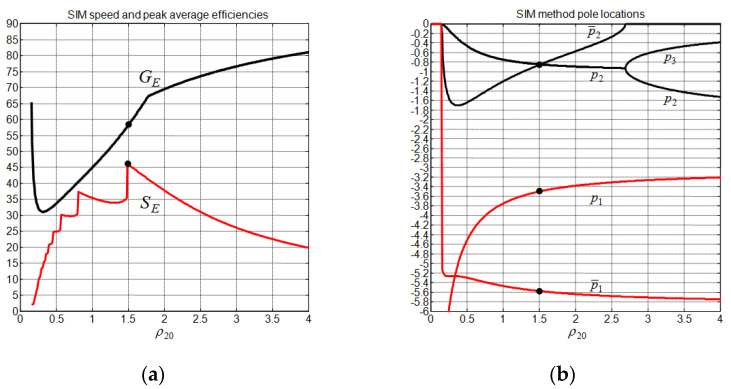
Analysis of the SIM method performance. (**a**) Speed and peak efficiencies; (**b**) pole locations.

For the proposed SIM method, one can impose a priori the system gain margin as a constraint to the controller. Using a gain margin of 1.63 and applying the proposed tuning method (43) with λ=1, we obtain $\rho_{10,g}=0.09$ and $\rho_{20,g}=1.5$. The obtained controller gains and their associated performances are also given in Table 2 and Table 3, respectively. Notice that the last assumption of Equation (2) is satisfied, since we have, for all controllers, $N_{1}>>mg$. In comparison to the other control methods, the proposed SIM method has the best pendulum angle deviation, cart undershoot, and control input effort at the price of degradation in the cart overshoot and settling time. Using the CPP for the linearized CIP system as an SSF, we obtained the best $G_{E}$ at a price of a slight degradation in $S_{E}$, as is indicated in Table 3. Figure 11 indicates that the obtained optimal SIM controller depends only on $S_{E}$, since we always have $S_{E}<G_{E}$. In addition to that, Table 4 and Figure 11 show that the optimal SIM controller is characterized by a set of two complex conjugates poles: one of them has a real part that is equal to the CPP controller pole, and the other one has the highest real part, which somewhat explains why the SIM controller has the largest cart settling time. Finally, Figure 10 tells us that the proposed SIM method allows to further reduce the peaking phenomenon while maintaining the system gain margin constant by increasing $\rho_{20}$ above 1.5 and maintaining $\rho_{10}=0.09$, but this enhancement is followed by an increase in $t_{cs}$. In the vicinity of $\rho_{20}=2.5$, the overshoot vanishes, and the cart settling time takes a value of about 4.8 s.

### 4.3. Performance Analysis without a Disturbance Input

**Figure 12:**
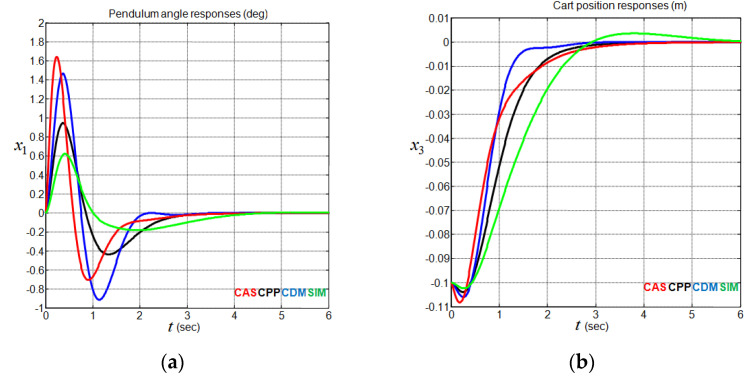
CIP output responses using CAS, CPP, CDM, and SIM methods. (**a**) Pendulum angle responses; (**b**) cart position responses.

**Figure 13:**
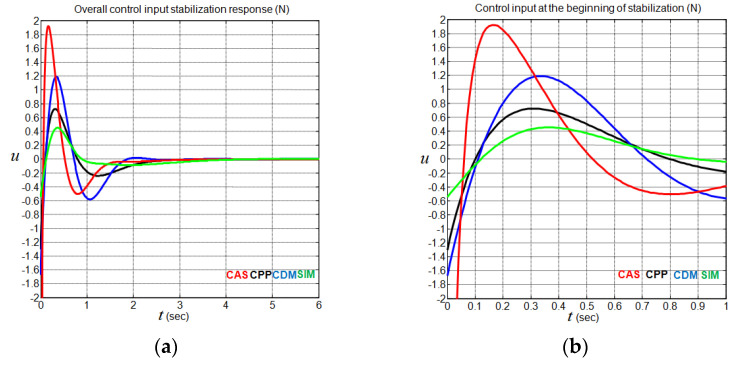
CIP input responses using CAS, CPP, CDM, and SIM methods. (**a**) Overall input responses; (**b**) restricted input responses.

To compare the performance of the above controllers, the reference cart position $x_{3d}$ is set to 0 m, and all the initial state values are set to zeros, except the initial cart position, which is set to $x_{3}\left(0\right)=-0.1$ m. The simulation results applied to the nonlinear CIP system (1), without considering disturbance input, are shown graphically in Figure 12 and Figure 13 and numerically in Table 5. One can notice at this point that all the assumptions given by Equation (2) are satisfied. The strong similarity that exists between the performance of the closed-loop linearized CIP system in Table 3 and the performance of the closed-loop nonlinear CIP system in Table 5 for each controller confirms the potential of linear control theory in solving the considered stabilization problem. On the other hand, Figure 12 shows that the SIM method exhibits good performance in the control input effort, the pendulum angle, and the cart position undershoot at the price of relatively large cart position overshoot and settling time. Concerning Figure 13, the fact that the control input effort demand occurs essentially at the beginning of the CIP system stabilization for all the considered controllers is obviously distinguished.

When the initial states begin from an equilibrium point and reach another one while the assumptions of Equation (2) are satisfied during the motion, the controlled nonlinear CIP system behaves similarly to the controlled linear CIP system. In such a situation, the performance of the controlled nonlinear CIP system can be naturally evaluated using the contour plots of Figure 6 and Figure 7 for a gain margin of 1.63 and given MPC parameters. However, when the states are far away from the equilibrium point, the classical prediction model, defined by Equations (7) and (8) together with k=+1, gives only partially consistent estimation or no longer holds at all. In the former case, which is not covered by the numerical study, it may perhaps be interesting to design the SIMO MPC controller in such a way to increase enough $\rho_{0}=\left(\rho_{10},\rho_{20}\right)$ in the hope to simultaneously increase the pendulum gains $N_{1}$ and $N_{2}$ while reducing the cart gains $N_{3}$ and $N_{4}$, as is suggested by Equations (26) and (29). When the pendulum angle is too large, the linearized model loses its validity, and it is necessary to apply some nonlinear technique to bring the pendulum to the vicinity of the upright equilibrium state. Then, it is possible to switch to the proposed control.

### 4.4. Performance Analysis with a Disturbance Input

**Figure 14:**
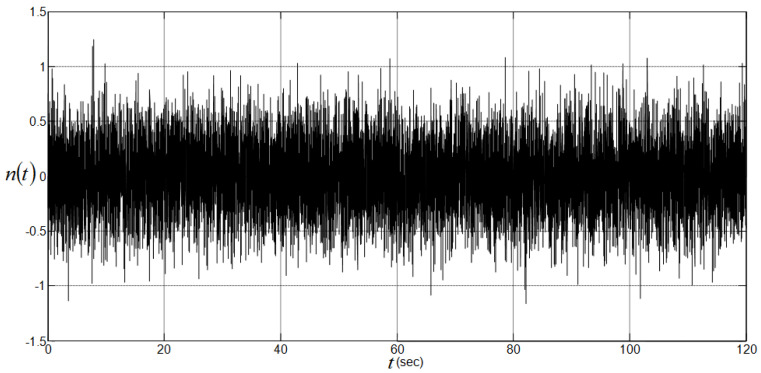
Input disturbance signal.

**Figure 15:**
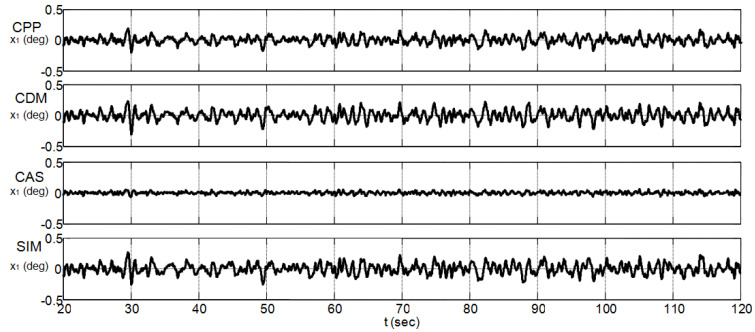
Pendulum angle responses with a disturbance input.

**Figure 16:**
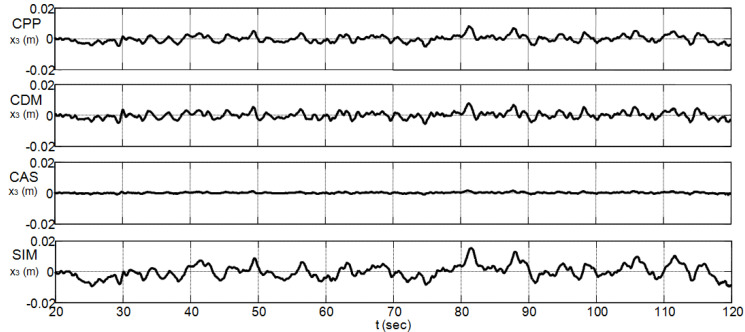
Cart position responses with a disturbance input.

To check the sensitivity of the studied controllers to disturbance input, a Matlab-Simulink band-limited white noise n(t) with sampling time 0.01 s, seed = 23,341, and power $P=10^{-3}$ was added to the control input signal of the nonlinear CIP system over a time interval of 120 s [23]. This disturbance input, which has an amplitude between −1.17 and 1.25, is depicted in Figure 14. Table 6 summarizes the resulting maximum absolute values of the CIP input and output signals that are obtained from the analysis of the last 100 s, i.e., in the assumed steady-state region. Regarding the disturbance input amplitude that appears in Figure 14, the controller performance of Table 5, and the maximum absolute values of Table 6, all the considered controllers appear to exhibit useful noise rejection capabilities. The CAS method, with its highest gains (see Table 2) and high transient control input effort (see Table 5), exhibits the best noise rejection capabilities in the steady-state region, whereas the proposed SIM method, with its lowest gains (see Table 2) and lowest transient control input effort (see Table 5), exhibits the worst one in the same steady-state region. Figure 15 and Figure 16 show graphically the obtained CIP output responses. Although the disturbance input level appears to dominate completely the amplitude of the SIM transient noise-free control effort, i.e., 0.54 N (see Table 5), the proposed SIM method still performs well even with a tuning method that does not take into account the presence of a disturbance input.

**Figure 17:**
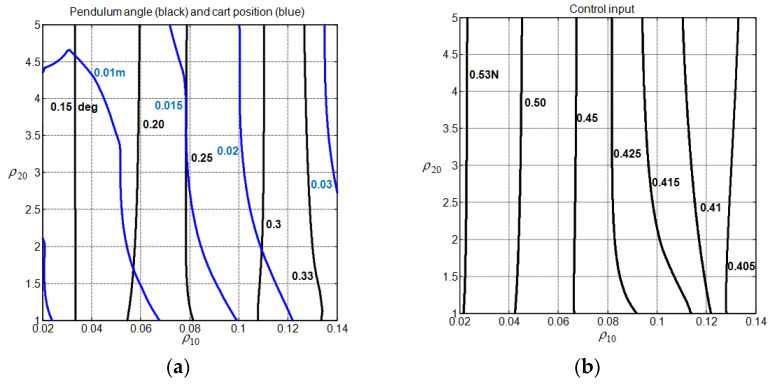
CIP steady-state performance with a disturbance input using SIM controllers. (**a**) Maximum absolute values of the pendulum angle and cart position; (**b**) maximum absolute values of the control input signal.

Let us now study the impact of the weight vector $\rho_{0}=\left(\rho_{10},\rho_{20}\right)$ on the closed-loop nonlinear CIP steady-state performance when the adopted disturbance input is considered. To this end, we keep $GM_{\min}=1.63$ and conduct 2500 simulations (obtained by using a coarse grid discretization with 50 points for $\rho_{10}$ in the interval $0<\rho_{10}<0.5$ and 50 points for $\rho_{20}$ in the interval $0<\rho_{20}<5$). Figure 17 shows the obtained contour plots for the maximum absolute values of the input and outputs nonlinear CIP system. It can be seen that the weight factor $\rho_{10}$ has a strong impact on $\sigma_{x1}$, $\sigma_{x3}$, and $\sigma_{u}$, while $\rho_{20}$ has only a moderate impact on $\sigma_{x3}$. In addition, one can clearly see that the increase in $\rho_{10}$ reduces the CIP input index $\sigma_{u}$, but as a side effect, it increases the CIP output indices $\sigma_{x1}$ and $\sigma_{x3}$.

**Remark 7.**  *This paper does not address the measurement noise issue explicitly. The experience with the cascade MPC controller in* [17] *suggests that low and moderate measurement noises are not problematic, and simple low-pass filtering can be used. This is also favored in* [33]. *However, high measurement noises present a challenge to the implementation of linear static state-feedback controllers. The problem lies in the need for differentiation of the pendulum angle and the cart position w.r.t. time. In such a situation, it is useful to consider sophisticated differentiations or other controller structures such as the output-feedback controller with or without observers to perform more efficient noise filtering at the price of an increase in controller design complexity*.

## 5. Conclusions

Most of the standard non-MPC linear static state-feedback controllers that are used to stabilize the nonlinear fourth-order CIP system around its desired equilibrium points are designed using pole-dependent techniques and without referring to a meaningful and transparent cost function. They are also prone to parameter tuning difficulties when dealing with the system transient performance and stability robustness simultaneously. To circumvent these difficulties, a linear static pole-independent MPC controller with only two tuning parameters is proposed. The controller ensures a priori a prescribed system gain margin and a two-time scale structure, allowing the response of the pendulum to be fast in comparison to the cart response. Using the linearized CIP model, some guidelines are developed in the form of two-dimensional contour plots for rapid MPC parameter tuning. Compared to the optimal CMD, CAS, and CPP methods, the proposed SIM method exhibits the best performance in the absence of disturbance input at the price of a slight degradation in the cart position overshoot and settling time. Future works will be devoted to studying the region of attraction associated with the proposed controller, reducing the sensitivity of the proposed method to disturbance input by optimizing the two-time-scale structure, relaxing the hypothesis of Assumption 1, and applying a similar method for other (linearized) robotic systems, e.g., the rotary inverted pendulum robot and the CIP with multiple links. The extension of the proposed method to deal directly with nonlinear system dynamics with or without uncertainties is also an interesting research topic.

## Figures and Tables

**Table 1:** Transient response performance on the UR and LR frontiers.

	*ρ*_10_	*ρ*_20_	*P*_*x*1*n*_	*V*_*x*3_ (%)	*D*_*x*3_ (%)	*P*_*un*_
*LR*	0.03 − 0.10	0.38 − 0.96	3.05 − 5.61	12.32 − 26.55	3.15 − 7.68	0.11 − 0.47
*UR*	0.02 − 0.16	0.87 − 3.23	0.97 − 2.80	0.36 − 6.31	0.82 − 3.61	0.02 − 0.22

**Table 2:** State-feedback controller gains and associated parameters.

	$N_{1}$	$N_{2}$	$N_{3}$	$N_{4}$	Control Parameter	GM
CPP	91.43	17.46	13.08	14.99	p=−3.49	1.62
CDM	82.08	14.87	16.35	14.81	τ=0.90	1.39
CAS	230.9	53.31	73.72	73.72	$\;h_{0}=0.20$	1.59
SIM	75.39	10.21	5.63	7.55	$\left(\rho_{10},\rho_{20}\right)\;=\left(0.09,1.5\right)$	1.63

**Table 3:** Controller performance comparison using linearized CIP model.

	$P_{x1}$ (deg)	$V_{x3}$ (%)	$D_{x3}$ (%)	$P_{u}\;\left(N\right)$	$t_{cs}\;(s)$	$S_{E}\;(\%)$	$G_{E}\;(\%)$
CPP	0.93	0.00	3.83	1.31	2.14	50.0	50.0
CDM	1.44	0.03	5.92	1.68	1.47	59.2	40.8
CAS	1.63	0.00	8.22	7.53	2.36	47.5	27.6
SIM	0.62	4.51	2.40	0.56	2.55	45.6	58.6

**Table 4:** Closed-loop poles for the considered controllers.

	$p_{1}+i{\bar{p}}_{1}$	$p_{2}+i{\bar{p}}_{2}$	$p_{3}+i{\bar{p}}_{3}$	$p_{4}+i{\bar{p}}_{4}$
CPP	−3.49	−3.49	−3.49	−3.49
CDM	−2.78−0.90i	−2.78−3.82i	−2.78+3.82i	−2.78+0.90i
CAS	−3.08−4.05i	−1.39	−23.77	−3.08+4.05i
SIM	−3.49−5.58i	−0.85−0.84i	−0.85+0.84i	−3.49+5.58i

**Table 5:** Controller performance comparison using nonlinear CIP model.

	$P_{x1}$ (deg)	$V_{x3}$ (%)	$D_{x3}$ (%)	$P_{u}\;\left(N\right)$	$t_{cs}$ (s)	$S_{E}$ (%)	$G_{E}$ (%)
CPP	0.95	0.00	3.88	1.31	2.16	49.8	49.7
CDM	1.47	0.00	5.99	1.68	1.42	60.1	40.5
CAS	1.64	0.00	8.26	7.53	2.37	47.4	27.5
SIM	0.62	3.65	2.37	0.54	2.55	45.6	60.6

**Table 6:** Performance in a noisy situation for 20 s ≤ *t* ≤ 120 s.

	CPP	CDM	CAS	SIM
*σ*_*x*1_ (deg)	0.2014	0.3107	0.0750	0.2647
*σ*_*x*3_ (m)	0.0080	0.0078	0.0016	0.0153
*σ*_*u*_ (N)	0.4271	0.4615	0.5623	0.4203

**Figure A1:**
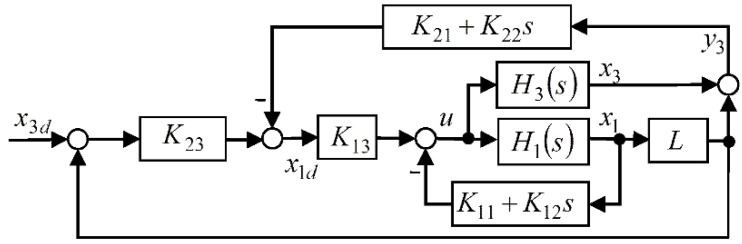
Cascade MPC controller for CIP system stabilization.

## Data Availability

The study did not report any data.

## Appendix A. The Standard CDM Controllers (CDM)

The CDM method is a general design tool that can be applied for example to a general fourth-order all-pole closed-loop system with the following transfer function:

(A1) $$G\left(s\right)=\frac{c_{0}}{c_{4}s^{4}+c_{3}\;s^{3}+c_{2}\;s^{2}+c_{1}s+c_{0}},$$

where $c_{0},c_{1},c_{2},c_{3},c_{4}$ are the coefficients of its the characteristic polynomial:

(A2) $$C\left(s\right)=c_{4}s^{4}+c_{3}\;s^{3}+c_{2}\;s^{2}+c_{1}s+c_{0}.$$

Using the CDM method, the characteristic polynomial of a fourth-order control system can be written in the following form [28]:

(A3) $$C\left(s\right)=\frac{1}{\gamma_{3}\gamma_{2}^{2}\gamma_{1}^{3}}\tau^{4}c_{0}s^{4}+\frac{1}{\gamma_{2}\gamma_{1}^{2}}\tau^{3}c_{0}\;s^{3}+\frac{1}{\gamma_{1}}\tau^{2}c_{0}\;s^{2}+\tau^{}c_{0}s+c_{0},$$

where $\gamma_{1},\gamma_{2},\gamma_{3}$ are the characteristic ratios, and τ is the GTC of the targeted closed-loop system. The set of adjusted parameters is then given by $\varphi =\left(\gamma_{1},\gamma_{2},\gamma_{3},\tau \right)$. It is well known that the step response of an all-pole system with such a characteristic polynomial has a small overshoot under the condition that the characteristic ratios are larger than 2 [28]. In addition, non-overshooting in the step response can be obtained using $\gamma_{1}\geq \gamma_{1LB}$ with $\gamma_{2}=\gamma_{3}=2$, where $\gamma_{1LB}=2.53$ is the minimum value of $\gamma_{1}$ that enable non-overshooting step responses. The standard CDM method, i.e., the Manabe form, is obtained by setting $\gamma_{1}=2.5$ and $\gamma_{2}=\gamma_{3}=2$. In this case, the settling time is about 2.5τ to 3τ, and for the fourth-order control system, all the poles are exactly on the vertical line, which means that the obtained two sets of complex conjugate poles have the same pole real part and different pole imaginary parts [31].

The design of an SFC using the CDM method for the CIP system can be conducted as follows. First, the cart transfer function $F_{3}\left(s\right)$ is transformed to the following form:

(A4) $$F_{30}\left(s\right)=-F_{3}\left(s\right)=\frac{s^{2}-a_{1}}{\frac{1}{b_{2}N_{3}}s^{4}+\frac{b_{1}N_{2}-b_{2}N_{4}}{b_{2}N_{3}}\;s^{3}+\frac{b_{1}N_{1}-b_{2}N_{3}-a_{1}}{b_{2}N_{3}}\;s^{2}+a_{1}\frac{N_{4}\;}{N_{3}}s+a_{1}}.$$

By equating the denominator of (A4) with the characteristic polynomial (A3) and using the standard CDM method, we obtain the following set of equations:

(A5) $$\begin{array}{l} b_{1}N_{2}-b_{2}N_{4}=\;\gamma_{3}\gamma_{2}\gamma_{1}\tau^{-1}=\;10\tau^{-1}\\ b_{1}N_{1}-b_{2}N_{3}-a_{1}\;=\gamma_{3}\gamma_{2}^{2}\gamma_{1}^{2}\tau^{-2}=50\tau^{-2}\\ N_{3}=a_{1}^{-1}b_{2}^{-1}\gamma_{3}\gamma_{2}^{2}\gamma_{1}^{3}\tau^{-4}=125a_{1}^{-1}b_{2}^{-1}\tau^{-4}\\ N_{4}=a_{1}^{-1}b_{2}^{-1}\gamma_{3}\gamma_{2}^{2}\gamma_{1}^{3}\tau^{-3}=125a_{1}^{-1}b_{2}^{-1}\tau^{-3} \end{array}.$$

Solving (A5) gives the SFC gains as follows:

(A6) $$\begin{array}{l} N_{1}=\;{\left(a_{1}b_{1}\right)}^{-1}\tau^{-4}\left(a_{1}^{2}\tau^{4}+50a_{1}\tau^{2}+125\right)\\ N_{2}=5{\left(a_{1}b_{1}\right)}^{-1}\tau^{-3}\left(2a_{1}\tau^{2}+25\right)\\ N_{3}=125a_{1}^{-1}b_{2}^{-1}\tau^{-4}\\ N_{4}=125a_{1}^{-1}b_{2}^{-1}\tau^{-3} \end{array},$$

where the GTC, i.e., τ, is the only parameter to be adjusted.

On the other hand, the transfer function (A4), which has two real zeroes, can be decomposed as follows:

(A7) $$\begin{array}{l} F_{30}\left(s\right)=\frac{s^{2}-a_{1}}{P\left(s\right)}=C_{1}\left(s\right)-C_{2}\left(s\right)\\ C_{1}\left(s\right)=\frac{s^{2}+a_{1}}{P\left(s\right)};\;\;\;C_{2}\left(s\right)=\frac{2a_{1}}{P\left(s\right)} \end{array},$$

where $C_{1}\left(s\right)$ is a non-all-pole system with one pair of jω-axis zeroes, and $C_{2}\left(s\right)$ is an all-pole system. Under the Manabe form, the authors of [28] showed the possibility to obtain a monotonic step response for $C_{1}\left(s\right)$ if τ is kept above a certain lower bound. However, obtaining the step response monotonicity for (A7) is almost impossible due to the presence of the NMP zero in this transfer function [29,30].

Now, combining (12), (22), and (A6) yields:

(A8) $$GM_{\min}=1+\frac{55a_{1}\tau^{2}}{2a_{1}^{2}\tau^{4}+50a_{1}\tau^{2}+1000}=1+\frac{55\tau_{0}^{2}}{2\tau_{0}^{4}+50\tau_{0}^{2}+1000},$$

where $\tau_{0}=a_{1}^{1/2}\tau$. The maximum system gain margin is equal to $0.5+2\sqrt{5}/5\approx 1.39$ at the normalized GTC $\tau_{0}=500^{1/4}\approx 4.73$.

## Appendix B. The Cascade MPC Controller (CAS)

In a previous paper [12], we have developed a two-horizon time cascade linear MPC controller to stabilize the CIP system. The controller scheme is composed of two MPC controllers in cascade. These controllers are tuned so as to obtain a doubly critically damped behavior for the inner and outer loops. For the purpose of a fair comparison, the cascade MPC (CAS) controller presented hereafter and shown in Figure A1 is an adapted version of the one developed in [12]. More precisely, to develop this version, we assume, as for the proposed SIMO MPC controller of this paper, that Assumption 1 is satisfied. In this case, the associated CAS pendulum prediction model, which is identical to the SIMO one, and the CAS cart prediction model, which is distinct from the SIMO one, are given hereafter:

(A9) $$\begin{array}{l} {\hat{x}}_{1}\;\left(t+h\right)=A_{1}x_{1}\left(t\right)+B_{1}x_{2}\left(t\right)\;-E_{1}u\\ {\hat{x}}_{2}\left(t+h\right)=A_{2}x_{1}\left(t\right)+B_{2}x_{2}\left(t\right)\;-E_{2}u \end{array}$$

(A10) $$\begin{array}{l} {\hat{x}}_{3}\;\left(t+h_{2}\right)=x_{3}\left(t\right)\;+h_{2}\;x_{4}\left(t\right)+0.5gx_{1d}\\ {\hat{x}}_{4}\left(t+h_{2}\right)=x_{4}\left(t\right)+gh_{2}x_{1d} \end{array},$$

where $\left(A_{i},B_{i},E_{i}\right)$ with i=1, 2 are evaluated using (8), and $x_{1d}$ is an unknown low-speed pendulum angle reference.

The cascade controller design can be conducted using two successive steps. In the first step, the design of the inner MPC controller $K_{1}=\left(K_{11},K_{12},K_{13}\right)$ is considered using the following quadratic cost function:

(A11) $$\begin{array}{l} J_{1}\left(t,h\right)=e_{1}^{2}\left(t+h\right)+\rho_{1}e_{2}^{2}\left(t+h\right)\\ e_{1}\left(t+h\right)=x_{1d}-{\hat{x}}_{1}\left(t+h\right)\\ e_{2}\left(t+h\right)=0-{\hat{x}}_{2}\left(t+h\right) \end{array}.$$

From the angle prediction model (A9), the control input is obtained by minimization of (A11) as follows:

(A12) $$\begin{array}{l} v_{0}\left(t\right)\;\;\;\;\;=-K_{11}x_{1}\left(t\right)-K_{12}x_{2}\left(t\right)+K_{13}x_{1d}\\ K_{11}=-\frac{A_{1}E_{1}+\rho_{1}A_{2}E_{2}}{E_{1}^{2}+\rho_{1}E_{2}^{2}};\;\;\;K_{12}=-\frac{B_{1}E_{1}+\rho_{1}B_{2}E_{2}}{E_{1}^{2}+\rho_{1}E_{2}^{2}};\;\;\;K_{13}=-\frac{E_{1}}{E_{1}^{2}+\rho_{1}E_{2}^{2}} \end{array}.$$

Imposing the critically damped constraint to the second-order inner loop gives [12]:

(A13) $$K_{11}=-a_{1}b_{1}^{-1}\left(d_{0}^{2}+4\right)/4;\;\;\;K_{12}=-a_{1}^{1/2}b_{1}^{-1}d_{0};\;\;\;K_{13}=-a_{1}b_{1}^{-1}d_{0}^{2}/4,$$

where $d_{0}=2a_{1}^{-1/2}\omega_{n0}\approx 1.2h_{0}^{-1}$ with the inner loop undamped natural frequency $\omega_{n0}\approx 0.6h^{-1}$.

In the second step, the design of the outer MPC controller $K_{2}=\left(K_{21},K_{22},K_{23}\right)$ is considered using a second quadratic cost function:

(A14) $$\begin{array}{l} J_{2}\left(t,h_{2}\right)=e_{3}^{2}\left(t+h_{2}\right)+\rho_{2}e_{4}^{2}\left(t+h_{2}\right)\\ e_{3}\left(t+h_{2}\right)=x_{3d}-{\hat{x}}_{3}\left(t+h_{2}\right)\\ e_{4}\left(t+h_{2}\right)=0-{\hat{x}}_{4}\left(t+h_{2}\right) \end{array}.$$

Using the cart prediction model (A10), the remaining control input is obtained by minimization of (A13) as follows:

(A15) $$\begin{array}{l} x_{1d0}\left(t\right)\;\;\;\;\;=-K_{21}x_{3}\left(t\right)-K_{22}x_{4}\left(t\right)+K_{23}x_{3d}\\ K_{21}=K_{23}=\frac{2g^{-1}}{h_{2}^{2}+4\rho_{2}};\;\;\;K_{22}=\frac{2h_{2}^{2}+4\rho_{2}}{h_{2}^{2}+4\rho_{2}}h_{2}^{-1}g^{-1} \end{array}.$$

Assuming that $K_{21}=K_{22}=K_{23}=K$ and $\omega_{n0}$ is high enough to obtain, the pole dominant principle leads to approximate the fourth-order cart transfer function by a simple second-order model [12]. Tuning the gain K to obtain a critically damped behavior for the outer loop leads to determining it with the following expression:

(A16) $$K=K_{21}=K_{22}=K_{23}=4/\left(4L+g\right).$$

From Figure A1, we get the following ECS:

(A17) $$u\left(t\right)=+N_{1}x_{1}\left(t\right)+N_{2}x_{2}\left(t\right)+N_{3}x_{3}\left(t\right)+N_{4}x_{4}\left(t\right)-N_{3}x_{3d},$$

with

(A18) $$\begin{array}{l} N_{1}=-K_{11}\\ N_{2}=-K_{12}-L\;KK_{13}\\ N_{3}=N_{4}=-KK_{13} \end{array},$$

where the cascade MPC controller gains are given in Equations (A13) and (A16).

Finally, let us notice here that by construction, the obtained cascade MPC gains are positive. The trivial prediction models, defined in (A9) and (A10), appear to be sufficient to conduct the design, and there is no need to define a generalized prediction model as in the case of the SIMO MPC method. Additionally, the evaluation of the optimal system gain margin for this controller leads to solving a fourth-order polynomial equation that is preferable to solve numerically.

## Appendix C. The Coincident Pole Placement Controller (CPP)

Taking into account the usefulness of driving the cart system with little or no oscillation to $x_{3d}$, a standard approach to design SFC controllers consist of determining the SFC controller gains $\left(N_{1},N_{2},N_{3},N_{4}\right)$ so as to obtain a coincident real negative pole p structure for the targeted closed-loop system $F_{3}\left(s\right)$ [17]. In this situation, the CIP characteristic polynomial follows the configuration $P\left(s\right)={\left(s-p\right)}^{4}$ that leads directly to evaluate the SFC controller gains by:

(A19) $$\begin{array}{l} N_{1}=b_{1}^{-1}a_{1}^{-1}p^{4}+6b_{1}^{-1}p^{2}+b_{1}^{-1}a_{1}\\ N_{2}=-4b_{1}^{-1}a_{1}^{-1}p^{3}-4b_{1}^{-1}p\\ N_{3}=a_{1}^{-1}b_{2}^{-1}p^{4}\\ N_{4}=-4a_{1}^{-1}b_{2}^{-1}p^{3} \end{array}.$$

The above controller can be viewed as optimal if its pole maximizes the gain margin (22). To this end, let us define the following normalized pole parameter:

(A20) $$p_{0}=a_{1}^{-1/2}p.$$

Then, from (31), (A19), and (A20), we obtain the set of algebraic equations:

(A21) $$\begin{array}{l} N_{10}D_{0}^{-1}=\;p_{0}^{4}+6p_{0}^{2}+1\\ N_{20}D_{0}^{-1}=-4p_{0}^{3}-4p_{0}\\ N_{30}D_{0}^{-1}=p_{0}^{4}\\ N_{40}D_{0}^{-1}=-4p_{0}^{3} \end{array}.$$

Now, substituting (31) in (22) and combining the obtained result with (A21) yields:

(A22) $$GM_{\min}=\frac{N_{20}D_{0}^{-1}\left(r_{12}-r_{34}\right)}{1+N_{40}D_{0}^{-1}\left(r_{12}-r_{34}\right)}=1+\frac{4p_{0}^{2}}{5p_{0}^{4}+2p_{0}^{2}+1}.$$

The maximum gain margin is equal to $0.5\left(1+\sqrt{5}\right)\approx 1.62$ at the pole location $p_{0}=-5^{-1/4}\approx -0.6687$. As it is stated in [17], the parameters (A19) that result from the above pole location choice allow one to tune the controller gains for any CIP system in the simplest fashion to ensure significantly improved performance and robustness. In this paper, we take this controller as a reference for the comparison task.

## Appendix D. Derivation of the Nonlinear CIP Dynamics

In this appendix, the basic equations for the CIP system, shown in Figure 1, are given as well as relevant explanation and derivation of the system of Equation (1). It is assumed that the pendulum rigid rod is mass-less, and the whole pendulum mass m is concentrated in the center of gravity of the pendulum ball. It is also assumed that no friction exists in the system between the cart and the track or between the cart and the pendulum. The coordinates of the center of the ball at the end of the pendulum, which are also the coordinates of the center of gravity of the mass-less pendulum, are given by:

(A23) $$\begin{array}{l} x_{G}=x_{3}+L\sin x_{1}\\ y_{G}=L\cos x_{1} \end{array}.$$

Now, Newton’s second law is applied to the cart to obtain:

(A24) $$M{\ddot{x}}_{3}=u-m{\ddot{x}}_{G}.$$

Then, substituting (A23) into (A24) and using the fact that $x_{2}={\dot{x}}_{1}$ and $x_{4}={\dot{x}}_{3}$ gives:

(A25) $$\begin{array}{ll} u & =M{\dot{x}}_{4}+m{\left({\dot{x}}_{4}+L\left[{\dot{x}}_{2}\cos x_{1}-x_{2}^{2}\sin x_{1}\right]\right)}^{}\\ & =\left(M+m\right){\dot{x}}_{4}-mLx_{2}^{2}\sin x_{1}+mL{\dot{x}}_{2}\cos x_{1} \end{array}.$$

From (A25), it is easy to get:

(A26) $${\dot{x}}_{4}=\frac{1}{M+m}\left[u+mLx_{2}^{2}\sin x_{1}-mL{\dot{x}}_{2}\cos x_{1}\right].$$

The rotational motion of the pendulum about its center of gravity involves two forces: the force due to gravity and the force due to the acceleration of the cart. Indeed, since the cart is moving, it applies a force on the pendulum. The moment of the force due to gravity is $mgL\sin x_{1}$, and the moment of the force due to the acceleration of the cart is $-mL{\dot{x}}_{4}\cos x_{1}$. The equation that describes the above motion is obtained by applying the rotational version of Newton’s second law. Summing the moments about the center of gravity of the pendulum, we obtain:

(A27) $$mL^{2}{\dot{x}}_{2}=mgL\sin x_{1}-mL{\dot{x}}_{4}\cos x_{1}.$$

From (A27), it is easy to get:

(A28) $${\dot{x}}_{2}=\frac{1}{mL^{2}}\left[mgL\sin x_{1}-mL{\dot{x}}_{4}\cos x_{1}\right].$$

Equations (A26) and (A28) describe the basic equations of the CIP system. Substituting (A26) into (A28) obtains:

(A29) $${\dot{x}}_{2}=-\frac{uL^{-1}\cos x_{1}-\left(M+m\right)gL^{-1}\sin x_{1}+mx_{2}^{2}\sin x_{1}\cos x_{1}}{M+m-m\cos^{2}x_{1}}.$$

Then, substituting (A29) into (A26) obtains:

(A30) $${\dot{x}}_{4}=+\frac{u-mg\sin x_{1}\cos x_{1}+mLx_{2}^{2}\sin x_{1}}{M+m-m\cos^{2}x_{1}}.$$

Finally, from ${\dot{x}}_{1}=x_{2}$, $x_{4}={\dot{x}}_{3}$, (A29), and (A30), we obtain the state-space format (1) of the nonlinear CIP dynamics.
